# Nonlinear KCCA in fMRI activation analysis: Self-supervised optimization and robust back-reconstruction

**DOI:** 10.1162/IMAG.a.1243

**Published:** 2026-06-04

**Authors:** Chendi Han, Zhengshi Yang, Xiaowei Zhuang, Dietmar Cordes

**Affiliations:** Cleveland Clinic Lou Ruvo Center for Brain Health, Las Vegas, NV, United States; Department of Psychology and Neuroscience, University of Colorado, Boulder, CO, United States

**Keywords:** data analysis, fMRI, task fMRI, activation, nonlinear kernel, CCA, KCCA

## Abstract

Recent studies have extended nonlinear kernels to Kernel Canonical Correlation Analysis (KCCA), enabling more flexible modeling of complex relationships globally. Building on these developments, we propose three key enhancements to nonlinear KCCA. First, inspired by self-supervised learning in machine learning research, we refine the parameter optimization process by adopting a subject-wise criterion designed to mitigate overfitting. Second, we introduce an improved back-reconstruction (inverse mapping) method that achieves higher accuracy and robustness than existing voxel-importance estimation methods. Third, we further investigate the kernel selection strategy based on convergence behavior, and validate its effectiveness through activation accuracy, data augmentation robustness, and eigendecomposition. The proposed framework is evaluated on both simulated and task-based fMRI datasets, with results demonstrating consistent improvements across multiple performance metrics.

## 1 Introduction

The task-fMRI activation detection problem aims to identify activation regions associated with a particular task design. From a methodological perspective, there are two primary ways to categorize existing methods. First, based on the input dimensionality, the methods can be classified as either local or global. Local methods, such as the General Linear Model (GLM) combined with a Gaussian Smoothing (GS) filter (Friman et al., 2003) or local Canonical Correlation Analysis (CCA) (Cordes et al., 2012; Friman et al., 2001; Yang et al., 2025; Zhuang et al., 2020), are computed in each local neighborhood around a voxel of interest. Global methods, such as Kernel Canonical Correlation Analysis (KCCA) and other techniques, detect activation in the entire brain in a single step (Han et al., 2025; Hardoon et al., 2004; Yang et al., 2018). Another classification considers whether the approach is linear or nonlinear. Linear methods, including GLM with GS (Friman et al., 2003), local CCA (Friman et al., 2001), and the linear KCCA (Hardoon et al., 2004; Yang et al., 2018), only capture linear relationships between the fMRI signal of the neural response and the hypothetical Hemodynamic Response Function (HRF), which is insufficient for HRF variability across different subjects or different voxel regions within a subject (Glover, 1999; Lindquist et al., 2009). Nonlinear approaches, such as local CCA with constraints (Cordes et al., 2012; Zhuang et al., 2020), deep CCA (Yang et al., 2025), or nonlinear KCCA (Han et al., 2025), can accommodate more general types of relationships.

One major challenge for global methods is the high dimensionality of unknowns relative to the available supervisory signals. Traditional task-fMRI data have a spatial dimension on the order of $10^{6}$, a time dimension on the order of $10^{2}$, and a limited number of subjects on the order of $10^{2}$. Nonlinear KCCA or classification problems involve estimating millions of unknowns with very few reference points. This imbalance can cause instability and overfitting in nonlinear methods. For example, in classification tasks, prior studies have shown that Support Vector Machines perform well with linear kernels, while nonlinear kernels are rarely used (Wang, 2009; Wang et al., 2007). Similar issues have been observed in decision tree-based models (Langs et al., 2011) and deep CCA (Xifra-Porxas et al., 2021). For the activation detection problem, when the activation pattern is treated as an unknown parameter with dimensionality equal to the number of voxels, even linear kernel methods without adequate regularization tend to overfit (Yang et al., 2018). Likewise, prior analyses indicate that certain nonlinear kernels can underperform compared with baseline methods in specific cases (Han et al., 2025).

To address overfitting, traditional methods often rely on additional datasets for supervision. For instance, some studies use task-fMRI data supplemented with simulated or pseudo-human data (Sorenson & Wang, 1996). Other approaches leverage resampled resting-state fMRI data (R. R. Nandy & Cordes, 2003, 2004), a technique also extended to linear KCCA, where the regularization parameter is chosen to maximize the difference between correlations in the task and resampled resting-state fMRI data (Laird et al., 2004; Yang et al., 2018). However, maximizing this difference does not yield optimal results as model complexity grows. Another disadvantage is that external datasets can be time consuming, and performance may depend on the choice of reference data. The second strategy is data augmentation. For example, a voxel-based shuffling method was employed in constrained local CCA to select the best parameter by maximizing robustness (Zhuang et al., 2017). However, this augmentation approach has only been applied to local methods.

Instead of relying on an external dataset for supervision, this study addresses the problem by focusing on the robustness of the activation patterns generated by nonlinear mappings. Similar approaches have been proposed in computer vision and medical imaging. A notable example is contrastive learning (T. Chen et al., 2020), introduced for pretraining Convolutional Neural Networks (CNNs). In this framework, augmented data are paired with original samples to form positive pairs (similar features) and negative pairs (dissimilar features). The CNN is then trained to maximize similarity within positive pairs and minimize similarity within negative pairs. Typically, augmented and original data are fed into the model simultaneously (T. Chen et al., 2020), forming a one-step approach. However, augmentation techniques such as cropping may inadvertently remove essential information. Another drawback is that blind augmentation without considering the underlying structure can introduce large randomness. As a result, contrastive learning often requires a very large batch size. To address this issue, augmentation methods based on previous outputs have been developed (Mo et al., 2021). This two-step approach is more closely related to the method we propose. Contrastive learning has also been extended to 3D medical imaging (Z. Zhou et al., 2021). Another self-supervised learning example is the vision transformer (Dosovitskiy et al., 2020), which divides images into patches and applies patch-wise augmentation. Further improvements, such as the pyramid structure in the Swin transformer, enhance segmentation performance by capturing fine details (Liu et al., 2021). These methods have been successfully adapted to medical image segmentation (J. Li et al., 2023; Tang et al., 2022) and connectivity detection (Bedel et al., 2023; B.-H. Kim et al., 2021; Yu et al., 2022).

In this study, we adopt the principle that an effective method should preserve prediction results under appropriate data augmentation. Building on this idea, we propose a novel augmentation technique that reshuffles voxel locations while preserving spatial correlations. We further validate this framework through analyses using both simulated and task-fMRI. Our results not only demonstrate that self-supervised learning works for fMRI activation detection problem but also explore the reasons behind its effectiveness (Zhang & Ma, 2022).

The second contribution of this study is a new back-reconstruction method. Because nonlinear kernels cannot be directly inverted (Wang et al., 2007; Yang et al., 2018), prior studies have estimated voxel importance to compute sensitivity maps in classification tasks using information calculated by the derivative. This approach originates from computer vision, where it is widely used for assessing pixel importance in image classification (Rasmussen et al., 2011; Selvaraju et al., 2017; Simonyan et al., 2013; Smilkov et al., 2017; B. Zhou et al., 2016). More recently, voxel-importance methods have been adapted for nonlinear KCCA in fMRI activation detection (Han et al., 2024, 2025). While effective across-subject average, this previous derivative-based method has several drawbacks: it often produces high subject-level variance, is computationally expensive for complex kernels, and relies on approximate derivatives, which limits accuracy. To overcome these limitations, we propose a more general inverse-mapping method that improves interpretability and computational efficiency, while reducing naturally to linear KCCA in special cases.

Finally, we address the problem of selecting appropriate kernel functions for fMRI analysis. While a strict mathematical proof is difficult to establish, several empirical approaches have been proposed. One approach is based on the convergence properties of kernel functions, with the general observation that bounded kernels often outperform unbounded ones. For example, unbounded kernels have been shown to cause failures in kernel principal component analysis (S. Y. Huang et al., 2009), whereas bounded kernels demonstrate greater robustness to noise and outliers in KCCA (Alam, 2014). Another promising direction is the use of multiple kernels. Prior work has demonstrated that mixed kernels, which combine bounded and unbounded functions, outperform single-kernel approaches in KCCA (Zhu et al., 2012). Similar strategies are widely used in computer vision, including architectures such as residual and densely connected networks (He et al., 2016; G. Huang et al., 2017). In nonlinear KCCA, a mixed hyperbolic tangent kernel—integrating linear, nonlinear, and bounded mappings—achieves state-of-the-art accuracy (Han et al., 2025). In this study, we further validate the effectiveness of these strategies from different perspectives. First, from a performance point of view, the mixed hyperbolic tangent kernel consistently achieves the highest performance across different datasets. Second, through independent data augmentation tests, such as noise contamination (Alam, 2014) or shuffling robustness, this kernel also demonstrates superior performance. Third, we analyze the eigendecomposition and find that bounded kernels, in general, capture more relevant features than unbounded ones.

This study is arranged as follows. In Section 2, we present two task-fMRI datasets and two simulated datasets used to cross-validate the method. Section 3 describes the pipeline for the nonlinear KCCA method, the self-supervised learning scheme, and the evaluation metrics. Section 4.1 summarizes the subject-level activation pattern and performance across-subject average for the two simulated and two task-fMRI datasets. Following the results, we further discuss the three main contributions of this study. The first, in Section 4.2, is the validation of our proposed self-supervised learning approach in several directions. The second, in Section 4.3, is a comparison of our newly proposed back-reconstruction method with previously published methods, as well as across different evaluation metrics. The third, in Section 4.4, compares the eigendecomposition results as further validation of the kernel selection. Section 4.5 discusses the activation overlays with regions-of-interest (ROI), which is more closely related to the neurological implications. Finally, in Section 5, we discuss the strengths of our method, comparisons with previous methods, and its limitations.

## 2 Data and Simulations

### 2.1 HCP dataset

Structural and functional MRI data were obtained from the Human Connectome Project (HCP) database (Van Essen et al., 2012), which includes 3T MRI data. We focus on the working memory task-fMRI study. Ninety-two male subjects aged 26–30 years were randomly selected, and the sample size is sufficient to test differences between methods. Five subjects were dropped during preprocessing due to missing information, yielding a final sample of 87 subjects. The task-fMRI data were acquired using a multiband factor of 8, TR/TE = 720/33.1 ms, FA = 52 degrees, 72 slices, spatial resolution = 2 mm×2 mm×2 mm , and in-plane size = 104 × 90. The first 15 timeframes were removed to avoid an unsaturated T1 signal.

The HCP working memory task (an n-back paradigm) includes three trial types: targets, non-targets, and lures. A target is a stimulus that satisfies the task-specific memory rule—for example, matching the item presented two trials earlier in the 2-back condition or matching the predefined cue in the 0-back condition. A non-target is a stimulus that does not meet this rule and, therefore, should not elicit a match response. A lure is a special type of non-target that closely resembles a target because it matches a nearby item in the sequence (e.g., the 1-back or 3-back item in a 2-back block), and thus has a higher likelihood of inducing a false-positive response.

As this study focuses on methodological development for activation analysis, we do not focus on gender-related activation patterns. Furthermore, since activation is evaluated at the subject level, we expect that even if such differences exist, they will not change the main results.

### 2.2 In-house scans

Eighteen subjects were originally consented and recruited. Two subjects with amnestic Mild Cognitive Impairment (aMCI) were excluded from the study due to incidental imaging abnormalities (meningioma and arachnoid cyst), yielding a final sample of 16 subjects. This dataset consists of eight subjects diagnosed with aMCI and eight cognitively normal controls (NC). Data acquisition was conducted with institutional review board approval using a 3T GE HDx MRI scanner equipped with an 8-channel head coil (Jin et al., 2012). The subjects in both groups were matched for age, education, and right-handedness. The detailed age and gender information are shown in Table 1. The acquisition parameters for the echo-planar imaging (EPI) sequence were: TR/TE = 2000 ms/30 ms, parallel imaging factor =2 , slices =25 (coronal oblique, perpendicular to the long axis of the hippocampus), slice thickness/gap =4.0 mm/1.0 mm, in-plane resolution 96×96 interpolated to 128×128 , yielding a voxel size of $1.72\times 1.72\times 5\;{\text{mm}}^{3}$. High-resolution structural images were also acquired including a standard T1-weighted image ($0.43\times 0.43\times 1\;{\text{mm}}^{3}$) and a coplanar standard T2-weighted image ($0.43\times 0.43\times 2.5\;{\text{mm}}^{3}$).

**Table 1.** Age and gender information for task-fMRI dataset.

	HCP dataset (NC)	In-house scans (NC)	In-house scans (MCI)
Number of subjects	87	8	8
Female	0	4 (50%)	3 (37.5%)
Age range in years	26–30	43–68	56–66

Each subject completed two episodic-memory tasks based on human faces and common objects, referred to as faces and pictures, respectively. Each episodic-memory paradigm included six periods. Each period comprised four phases: encoding, distraction, recognition, and a short instruction prompt reminding participants of the upcoming task. During the encoding phase, participants viewed seven face–occupation pairs presented sequentially, each for 3 seconds (21 seconds total), and were asked to memorize them. This was followed by an 11-second distraction phase, during which participants were instructed to respond as quickly as possible by pressing the right button when the letter “Y” appeared and the left button when the letter “N” appeared on the screen. The recognition phase then presented 14 items—7 repeats from the encoding phase and 7 novel face–occupation pairs. Participants responded with the right button if the stimulus had been seen previously and the left button if it was new. For each episodic-memory task, the total scan duration was 9 minutes and 36 seconds, yielding 288 volumes. The first 10 seconds (5 timeframes) were removed to avoid an unsaturated T1 signal.

### 2.3 Contrast and effective design signal

The HCP working memory task includes three different trial types: targets, non-targets, and lures. Each is encoded as a binary time series, which is then convolved with a canonical HRF to generate a design matrix represented as $\text{X}\in {ℜ}^{390\times 3}$ . This study uses the contrast targets minus non-targets $\text{C}={\left[1,-1,\;0\right]}^{T}$. This contrast isolates neural activity specifically related to working memory matching and decision processes by subtracting baseline perceptual and response-related effects that are common to both trial types.

For the in-house episodic memory scans, the paradigm includes four trial types: instruction, control, encoding, and recognition. Each event type is encoded as a binary time series and convolved with a canonical HRF, yielding a design matrix $\text{X}\in {ℜ}^{283\times 4}$ . Two contrasts are then defined: Encoding minus Control (EC, $\text{C}={\left[0,-1,\;1,\;0\right]}^{T}$) and Recognition minus Control (RC, $\text{C}={\left[0,-1,0,1\right]}^{T}$). The EC contrast isolates neural activity specifically related to memory formation by removing low-level perceptual and motor components shared with the control condition, while the RC contrast highlights retrieval-related activity after similar removal of baseline task demands (Jin et al., 2012).

For a specific contrast C, the effective design signal ${\text{X}}_{\text{eff}}$ is generated according to the method described in Cordes et al. (2012)

$${\text{X}}_{\text{eff}}=\text{X}{\left({\text{X}}^{T}\text{X}\right)}^{-1}\text{C}{\left[{\text{C}}^{T}{\left({\text{X}}^{T}\text{X}\right)}^{-1}\text{C}\right]}^{-1}.$$ (1)

Next, we map ${\text{X}}_{\text{eff}}$ into the linear kernel space using the equation $K_{\text{X}}={\text{X}}_{\text{eff}}{\text{X}}_{\text{eff}}^{T}$. Subsequently, we focus on the effective design signal.

### 2.4 General framework for simulated data generation

Besides task-fMRI, simulated fMRI also provides valuable information for the activation detection problem as the ground truth is given. We generate simulated data using

$$\begin{array}{cc} \begin{array}{c} {\text{Y}}_{\text{simulated}}={\text{Y}}_{\text{noise}}+\rho {\text{Y}}_{\text{signal}},\\ {\left({\text{Y}}_{\text{signal}}\right)}_{q}=\{\begin{array}{c} \;\;{\text{X}}_{\text{add}},\;\;\;for\;q\in M\\ 0,\;\;\;for\;q\notin M \end{array} \end{array} & \end{array}$$ (2)

where T denotes the length of time, Q represents the number of voxels, M is a mask that contains all activated voxels, ${\left({\text{Y}}_{\text{signal}}\right)}_{q}\in {ℜ}^{T\times 1}$ is equal to the added signal ${\text{X}}_{\text{add}}$ if voxel q is activated, ${\text{Y}}_{\text{noise}}$ is the noise considered independent to ${\text{X}}_{\text{add}}$ , and ρ is the signal strength. Numerically, ${\text{Y}}_{\text{noise}}$ and ${\text{X}}_{\text{add}}$ are normalized in time with a mean of 0 and a variance of 1. The choice for these parameters is dataset specific as listed in the following sections. A consistent feature across all simulations is that M includes approximately 10% of the total number of voxels in the brain.

### 2.5 fMRI simulation 1

**Algorithm 1.** Procedure for determining the signal strength ρ with baseline-consistent performance

Generate ${\text{Y}}_{\text{signal}}$ , ${\text{Y}}_{\text{noise}},$ and ${\text{X}}_{\text{eff}}$ . Start with ρ=0.1 **while True do** Compute the receiver operating characteristic based on the general linear model with Gaussian smoothing (Section 3.3) Compute the Area Under the Curve (AUC) for a false positive rate (FPR) smaller than 0.1 **if** ${\text{AUC}}_{\text{FPR}<0.1}<0.035$ **then** ρ=ρ+0.005 **else if** ${\text{AUC}}_{\text{FPR}<0.1}>0.05$ **then** ρ=ρ−0.005 **else** **break** **end if** **end while**

**Fig. 1.**
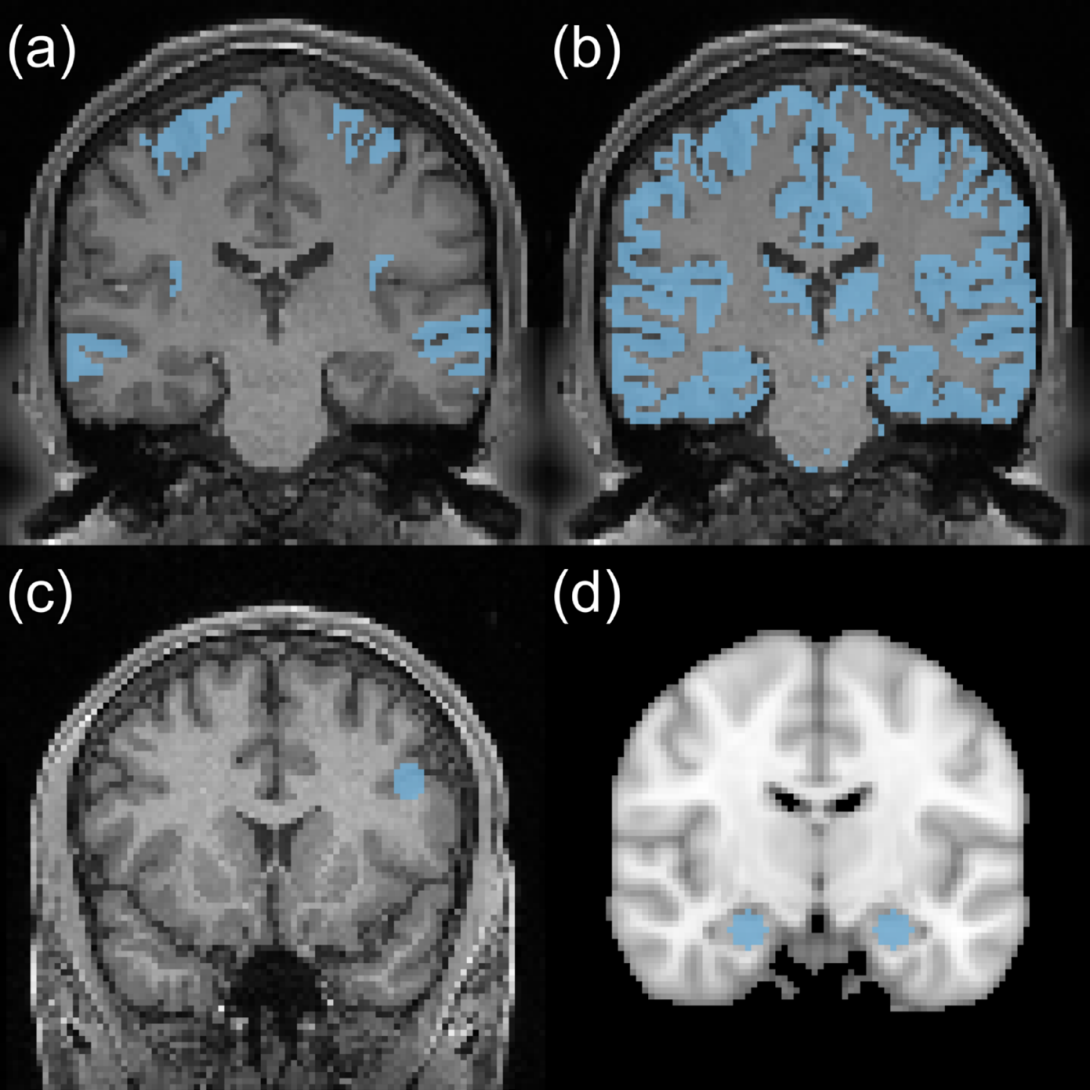
Ground truth or desired activation location overlaid on the T1 image. (a) Illustration of the ground truth mask for the first simulated fMRI dataset. Activations are shown in the precentral gyrus (upper) and the middle temporal gyrus (lower). (b) Illustration of the gray matter; the subject is the same as in (a). (c) Illustration of the working memory regions of interest (ROI), with a spherical center located at MNI coordinates (−44,14,29) in the left prefrontal cortex and a radius of 6 mm, overlaid on the T1 image from one subject in the HCP dataset. (d) Illustration of the episodic memory ROI, with two spheres centered at MNI coordinates (24,−12,−20) and (−24,−12,−20) in the hippocampus, each with a radius of 6 mm, overlaid on the T1 image from one subject in the in-house scans.

The key components for the first simulated fMRI dataset are listed below:

1. Mask M: The activation mask is selected for specific brain regions. We focus on six bilateral Automated Anatomical Labelling (AAL) regions (Tzourio-Mazoyer et al., 2002), including the anterior cingulate cortex, precentral gyrus, inferior frontal gyrus, insula, middle frontal gyrus, and middle temporal gyrus, which are treated as active. In total, approximately 10% of voxels in the brain (or around 19% of voxels in the gray matter) are activated. Let $M\in {ℜ}^{1\times Q}$ be the mask that equals 1 for voxels belonging to chosen AAL regions and 0 otherwise (Tzourio-Mazoyer et al., 2002). An example of the activation is shown in Figure 1a.
2. Noise ${\text{Y}}_{\text{noise}}$ : The resampled resting-state fMRI data are considered noise, as it does not contain task-related information. Since the time dimension for the resampled resting-state fMRI data in the HCP dataset is higher than that for the working memory task, we select the timeframes between 301 and 690 to match the time dimension.
3. Effective design signal ${\text{X}}_{\text{eff}}$ : Given three different event types: targets, non-targets, and lures contrasts, we use the contrast targets minus non-targets $\text{C}={\left[1,-1,\;0\right]}^{T}$ to generate the effective design signal.
4. Added signal ${\text{X}}_{\text{add}}$ : We assume that the real signal does not exactly match the effective design signal. In other words, ${\text{X}}_{\text{add}}$ is not exactly the same as ${\text{X}}_{\text{eff}}$ . It is generated based on the following: first perturb the original contrast by ${\text{C}}^{\prime }={\left[1,-1,\;0\right]}^{T}+0.1N\left(\text{0},\text{I}\right)$, then use Eq. (1) ${\text{X}}_{\text{add}}=\text{X}{\left({\text{X}}^{T}\text{X}\right)}^{-1}{\text{C}}^{\prime }{\left[{\left({\text{C}}^{\prime }\right)}^{T}{\left({\text{X}}^{T}\text{X}\right)}^{-1}{\text{C}}^{\prime }\right]}^{-1}$ . Here, N(0,I) refers to the normal distribution.
5. Signal strength ρ: Along with the noise, activation mask, added signal, and effective design signal, the simulated data and ground truth are generated using Eq. (2). The signal strength ρ is computed using Algorithm 1. Specifically, starting from ρ=0.1 , the algorithm will evaluate the performance from the General Linear Model with Gaussian smoothing (GLM+GS) method described in Section 3.3 and compute the Receiver Operating Characteristic (ROC) curve. The value of ρ is adjusted—either increased or decreased—such that the Area Under the Curve (AUC) for False Positive Ratio (FPR) smaller than 0.1 falls within the range of 0.035 to 0.05 . This algorithm ensures that the signal strength is neither too high nor too low. Similar methods have been adopted in Yang et al. (2020) to reduce the type 1 error in fMRI data analysis. The exact value is shown in Table 2.
6. Spatial smoothing: For the baseline model GLM+GS, a Gaussian smoothing filter with fixed Full Width at Half Maximum (FWHM) of 4 mm is applied to ${\text{Y}}_{\text{simulated}}$ (Yang et al., 2018). The correlation is computed between ${\text{X}}_{\text{eff}}$ and ${\text{Y}}_{\text{simulated}}$ after the smoothing. For the kernel method, seven steerable filters generated from FWHM =4 mm are applied to the dataset (Yang et al., 2018). Detailed information about the smoothing kernels is provided in Appendix A.1.

**Table 2.** Summary of noise, signal strength, and mask for the simulation data.

	${\text{Y}}_{\text{noise}}$	ρ	M
fMRI simulation 1	Resting-fMRI data	0.0446±0.0123	Six selected AAL regions
fMRI simulation 2	Resting-fMRI data	0.2624±0.1279	10 % of voxels with high correlation to ${\text{X}}_{\text{eff}}$
1D simulation	Gaussian white noise	0.3	Randomly chosen 10 % of all voxels

Compared with previous simulation methods, our approach introduces several key differences. In contrast to Yang et al. (2020), where different contrasts were applied to different brain regions, we use a single contrast with relatively small perturbations. While this sacrifices spatial heterogeneity, it ensures that each cluster receives the same signal strength, making it easier to assess the effects of kernel-based activation detection methods in a controlled setting. Compared with Han et al. (2025), we retain the sign of α to be consistent in this study. Our practical experience suggests that for relatively small nonlinear effects, preserving the sign leads to more accurate results under the same signal strength. Another important difference is that since several kernels exhibit nearly identical performance, we increase the number of simulated subjects from 20 to 50 to obtain more stable results and reduce variance in performance evaluation.

### 2.6 fMRI simulation 2

The simulation method is discussed in Section 2.5 and still includes specific signals that are manually added. One concern is that the added signal may differ from real signals. We consider an alternative approach for adding the signal. We still treat the noise as resampled resting-state fMRI data, which is assumed to be unrelated to the task, but modify the way the signal is added. Specifically, for the task-fMRI data, a uniform Gaussian smoothing filter with FWHM =4 mm is applied, and contrasts using targets minus non-targets $\left(\text{C}={\left[1,-1,\;0\right]}^{T}\right)$ are used to generate ${\text{X}}_{\text{eff}}$ . After the smoothing filter, voxels with the top 1% correlation to ${\text{X}}_{\text{eff}}$ are assumed to be most likely activated. Task-fMRI data from these voxels are extracted, their signals are randomly shuffled, and the shuffled signals are added to regions with the top 10% of correlations (Cordes et al., 2012). This approach aims to generate a more realistic added signal rather than one derived solely from the hypothetical HRF.

1. Mask M: For task-fMRI, find voxels with top 10% of correlations to the effective design signal after GLM+GS as the mask. An example of the activation is shown in Figure 5a.
2. Noise ${\text{Y}}_{\text{noise}}$ : Choose resampled resting-state fMRI data with a timeframe between 301 and 690 .
3. Effective design signal ${\text{X}}_{\text{eff}}$ : Use contrast targets minus non-targets $\text{C}={\left[1,-1,\;0\right]}^{T}$ from the HCP working memory task to generate the effective design signal.
4. Added signal ${\text{X}}_{\text{add}}$ : Choose task-fMRI from voxels with top 1% of correlations to the effective design signal after GLM+GS. As a note, the smoothing is only used to compute correlation, so the added signal is not smoothed.
5. Signal strength ρ: The signal strength ρ is computed using Algorithm 1. The exact value is shown in Table 2.
6. Spatial smoothing: Following the setup of the first simulation, GLM with GS (FWHM =4 mm) serves as the baseline. The kernel method employs seven steerable filters constructed with FWHM =4 mm and applied to ${\text{Y}}_{\text{simulate}}$ .

For a similar simulation, we compare our approach with Cordes et al. (2012). In the previous work, the “true activation” was defined using voxels with a statistical threshold of $p=10^{-6}$ . In contrast, we define activation based on the top 1% of voxels with the highest activation values. Regarding the mask selection, we use the top 10% of voxels that exhibit the strongest correlation with the design signal. This choice ensures that the number of activated voxels is roughly the same as in our first simulation. In contrast, Cordes et al. (2012) used a two-dimensional grid structure for the mask.

### 2.7 One-dimensional simulation

Consider a one-dimensional (1D) system where q represents a specific location, $Q=10^{4}$ is the total spatial length, and T=100 is the total number of time points.

1. Mask M: Randomly choose 10% of locations q∈M .
2. Noise ${\text{Y}}_{\text{noise}}$ : Choose Gaussian white noise with mean 0 and standard deviation 1.
3. Effective design signal ${\text{X}}_{\text{eff}}$ : Define ${\text{X}}_{\text{eff}}=\text{sin}\left(t+\phi \right)$ with t∈[0, 2π] and ϕ is a uniform random number with ϕ∈(−π/4, π/4).
4. Added signal ${\text{X}}_{\text{add}}$ : ${\text{X}}_{\text{add}}=\text{sin}\left(t\right)$.
5. Signal strength ρ: ρ=0.3 . Under the given signal strength, the AUC with FPR <0.1 typically ranges from 0.04 to 0.06 .
6. Spatial smoothing: No smoothing is performed.

## 3 Methods

### 3.1 fMRI preprocessing

The fMRI data were minimally preprocessed using the SPM12 package (Ashburner, 2009), including realignment, slice-timing correction, coregistration, and normalization to MNI atlas (Glasser et al., 2013). A high-pass filter with a cutoff frequency of 1/120 Hz was then applied to remove temporal drift, as commonly recommended by fMRI preprocessing software such as SPM or fMRIPrep (Esteban et al., 2019; Frackowiak et al., 2004). No spatial smoothing was performed in this stage. Finally, the data were normalized to have a temporal mean of 0 and a variance of 1. We represent the preprocessed task-fMRI data as $\text{Y}\in {ℜ}^{T\times Q}$ , where T denotes the length of time and Q represents the number of voxels. Although realignment (which internally performs motion correction) was applied, motion parameters were not used as regressor (Behzadi et al., 2007), as we aimed to avoid introducing high-frequency artifacts (J. E. Chen et al., 2017; Yakupov et al., 2017).

### 3.2 Steerable filters

To reduce spatial blurring, steerable filters are employed for all kernel-related analyses. Specifically, seven steerable filters generated from a Gaussian kernel with FWHM of 4 mm are used (Yang et al., 2018). The equally weighted summation of these seven filters is equivalent to a single Gaussian filter with FWHM = 4 mm. A detailed description of the steerable filters is provided in Appendix A.1.

### 3.3 General linear model with Gaussian smoothing filter

We use the General Linear Model (GLM) with a Gaussian Smoothing (GS) filter as a baseline model for all kernel methods. The Gaussian filter is selected to achieve the same level of smoothing as the steerable filters. The correlation is then evaluated between the smoothed fMRI data and ${\text{X}}_{\text{eff}}$ .

### 3.4 Linear and nonlinear kernels

For the fMRI signal, we first implement a linear mapping of the data from Y to the feature space $\tilde{\text{Y}}$ by $\tilde{\text{Y}}=\text{YA}\in {ℜ}^{T\times P},$ where $\text{A}\in {ℜ}^{Q\times P}$ is the spatial transformation matrix, T is the number of time points, Q is the number of voxels, and P is the number of voxels after spatial transformation. For example, standard GS can be done by one isotropic Gaussian filter function so that P=Q (Friman et al., 2003). More recent work uses 7 3D steerable filters such that P=7Q (Yang et al., 2018). After smoothing, $\tilde{\text{Y}}$ is input to eight different kernels, as shown in Table 3, to map the data from the original space to the kernel spaces.

**Table 3.** Summary of eight kernels, parameter constraints, and optimization steps.

Kernels	Expression	Parameters	Steps
Linear	$K_{\text{Y}}\left({\tilde{\text{Y}}}_{i},{\tilde{\text{Y}}}_{j}\right)={\tilde{\text{Y}}}_{i}{\tilde{\text{Y}}}_{j}^{T}$	$\gamma \in \left(0.1,\;10^{4}\right)$	15
Parabolic	$K_{\text{Y}}\left({\tilde{\text{Y}}}_{i},{\tilde{\text{Y}}}_{j}\right)={\left({\tilde{\text{Y}}}_{i}{\tilde{\text{Y}}}_{j}^{T}+b^{2}\right)}^{2}$	$b^{2}\in \left(0,\;1\right)$ and $\gamma \in \left(0.1,\;10^{4}\right)$	30
Gaussian	$K_{\text{Y}}\left({\tilde{\text{Y}}}_{i},{\tilde{\text{Y}}}_{j}\right)=\text{exp}\left(-∥{\tilde{\text{Y}}}_{i}-{\tilde{\text{Y}}}_{j}{∥}^{2}/\sigma^{2}\right)$	$\sigma^{2}\in \left(0.1,\;10\right)$ and $\gamma \in \left(0.1,\;10^{4}\right)$	30
Inverse	$K_{\text{Y}}\left({\tilde{\text{Y}}}_{i},{\tilde{\text{Y}}}_{j}\right)=1/\sqrt{∥{\tilde{\text{Y}}}_{i}-{\tilde{\text{Y}}}_{j}{∥}^{2}+b^{2}}$	$b^{2}\in \left(0.1,\;1\right)$ and $\gamma \in \left(0.1,\;10^{4}\right)$	30
Bounded Linear	$K_{\text{Y}}\left({\tilde{\text{Y}}}_{i},{\tilde{\text{Y}}}_{j}\right)=\text{min}\left(C,{\tilde{\text{Y}}}_{i}{\tilde{\text{Y}}}_{j}^{T}\right)$	C∈(0.1, 10) and $\gamma \in \left(0.1,\;10^{4}\right)$	30
Square	$K_{\text{Y}}\left({\tilde{\text{Y}}}_{i},{\tilde{\text{Y}}}_{j}\right)=∥{\tilde{\text{Y}}}_{i}-{\tilde{\text{Y}}}_{j}{∥}^{2}$	$\gamma \in \left(0.1,\;10^{4}\right)$	15
Tanh	$K_{\text{Y}}\left({\tilde{\text{Y}}}_{i},{\tilde{\text{Y}}}_{j}\right)=\text{tanh}\left(b{\tilde{\text{Y}}}_{i}{\tilde{\text{Y}}}_{j}^{T}+c\right)$	b,c∈(−1, 1), and $\gamma \in \left(0.1,\;10^{4}\right)$	60
Mixed Tanh	$K_{\text{Y}}\left({\tilde{\text{Y}}}_{i},{\tilde{\text{Y}}}_{j}\right)=\text{tanh}\left(b_{1}{\tilde{\text{Y}}}_{i}{\tilde{\text{Y}}}_{j}^{T}+b_{2}∥{\tilde{\text{Y}}}_{i}-{\tilde{\text{Y}}}_{j}{∥}^{2}+c\right)$	$\left\{b_{1},b_{2},c\right\}\in \left(-1,\;1\right)$ and $\gamma \in \left(0.1,\;10^{4}\right)$	200

KCCA represents fMRI data through a matrix $K_{\text{Y}}\in {ℜ}^{T\times T}$ . The traditionally used linear kernel can be written as $K_{\text{Y}}\left({\tilde{\text{Y}}}_{i},{\tilde{\text{Y}}}_{j}\right)={\tilde{\text{Y}}}_{i}{\tilde{\text{Y}}}_{j}^{T}$, where ${\tilde{\text{Y}}}_{i}$ represents the smoothed fMRI data at time i, with the diagonal element ${\left(K_{\text{Y}}\right)}_{ii}=\sum_{q}{\tilde{\text{Y}}}_{iq}{\tilde{\text{Y}}}_{iq}$ (Hardoon et al., 2004). In fMRI analysis, where noise levels are much higher than signal strengths, this operation generates large diagonal elements due to the noise self-correlation effect. This issue cannot be mitigated by centralized methods (Bengio et al., 2004; B. Chen et al., 2017). Instead of self-correlation, previous fMRI data have shown that noise correlation effects can occur at different times (Cordes & Nandy, 2007; X. Li, 2014). The noise correlation in fMRI (including self-correlation) can produce large matrix elements with no relationship to the signal, potentially making KCCA unstable.

We consider two approaches to mitigate noise correlation effects. The first approach involves computing the difference. If we define $K_{\text{Y}}\left({\tilde{\text{Y}}}_{i},{\tilde{\text{Y}}}_{j}\right)=∥{\tilde{\text{Y}}}_{i}-{\tilde{\text{Y}}}_{j}{∥}^{2}$, the diagonal components are entirely removed by this operation. The second approach avoids high values through nonlinear mappings, such as a bounded linear function or a hyperbolic tangent function. These two approaches can be combined using a mixed hyperbolic tangent kernel. An additional advantage of the hyperbolic tangent function is its ability to revert to a linear kernel. We later observe that this property enhances stability across different tasks and datasets, making it a more robust choice for fMRI analysis.

We also examine the effect of increasing the noise self-correlation effect in matrix elements, such as introducing a parabolic kernel constructed by taking another square over a linear kernel. This further increases the difference between the diagonal and off-diagonal elements.

For other kernels, such as Gaussian or inverse square root (Fasshauer, 2007; Salazar et al., 2024), which map the matrix elements inversely, our findings indicate that although they roughly maintain the matrix elements within a similar range and their mapping functions can mitigate the large positive values generated by noise self-correlation, they generally do not yield significant improvements over the linear kernel.

In summary, we choose eight different kernels as defined in Table 3. We divide these kernels into four groups based on the function convergence and correlation properties with respect to the input. Group 1: Linear and parabolic kernels. Both are unbounded for positive and negative infinity. Group 2: Gaussian kernel and inverse square root kernel. Both are bounded in the positive region but cannot be approximated by the linear kernel. In the latter, we will abbreviate the inverse square root kernel as the inverse kernel. Group 3: Bounded linear and square kernels. These are not fully bounded functions but are designed specifically to mitigate the noise self-correlation effect. Group 4: Hyperbolic tangent and mixed hyperbolic tangent kernels. These are fully bounded functions for both positive and negative regions. We abbreviate them as tanh and mixed tanh in the latter.

All kernels in Table 3 can be generated using linear and square kernels. For these two kernels, the data are normalized to their mean absolute values to ensure consistent data ranges. A detailed formula is shown in Appendix A.2. After nonlinear mapping, the matrix value range can change, particularly the parabolic kernel or hyperbolic tangent kernel when b is small. We perform an additional normalization to ensure consistent data ranges, which improves numerical stability when computing correlations in the kernel space. We can show that the hyperbolic tangent can approximate a linear function when b is small and c=0 , and the mixed hyperbolic tangent can approximate a linear or square function when $b_{1}$ or $b_{2}$ is small and c=0 . Theoretically, a bounded linear kernel can also approximate a linear function when C is large, but due to the highly unbalanced matrix elements, this relationship is hard to observe in our numerical study.

In nonlinear KCCA, we keep $K_{\text{X}}$ as a linear kernel for two reasons. First, the nonlinear mapping can in principle be applied either to the observed fMRI data or to the hypothesized HRF regressors. In this study, we apply the nonlinear transformation only to the data side while keeping the HRF regressors linear. This choice follows a common practice in machine learning, where nonlinear feature mappings are typically applied to the input data rather than to the target variables. For example, in neural network models such as CNNs, nonlinear transformations are introduced to the data features, whereas the labels remain unchanged. Second, from a mathematical perspective, applying nonlinear mappings to both sides is often equivalent to applying a single nonlinear transformation while keeping the other side linear. Therefore, following the conventional practice, we introduce the nonlinear mapping only to the data in the present study.

### 3.5 Compute correlation in the kernel space

**Fig. 2.**
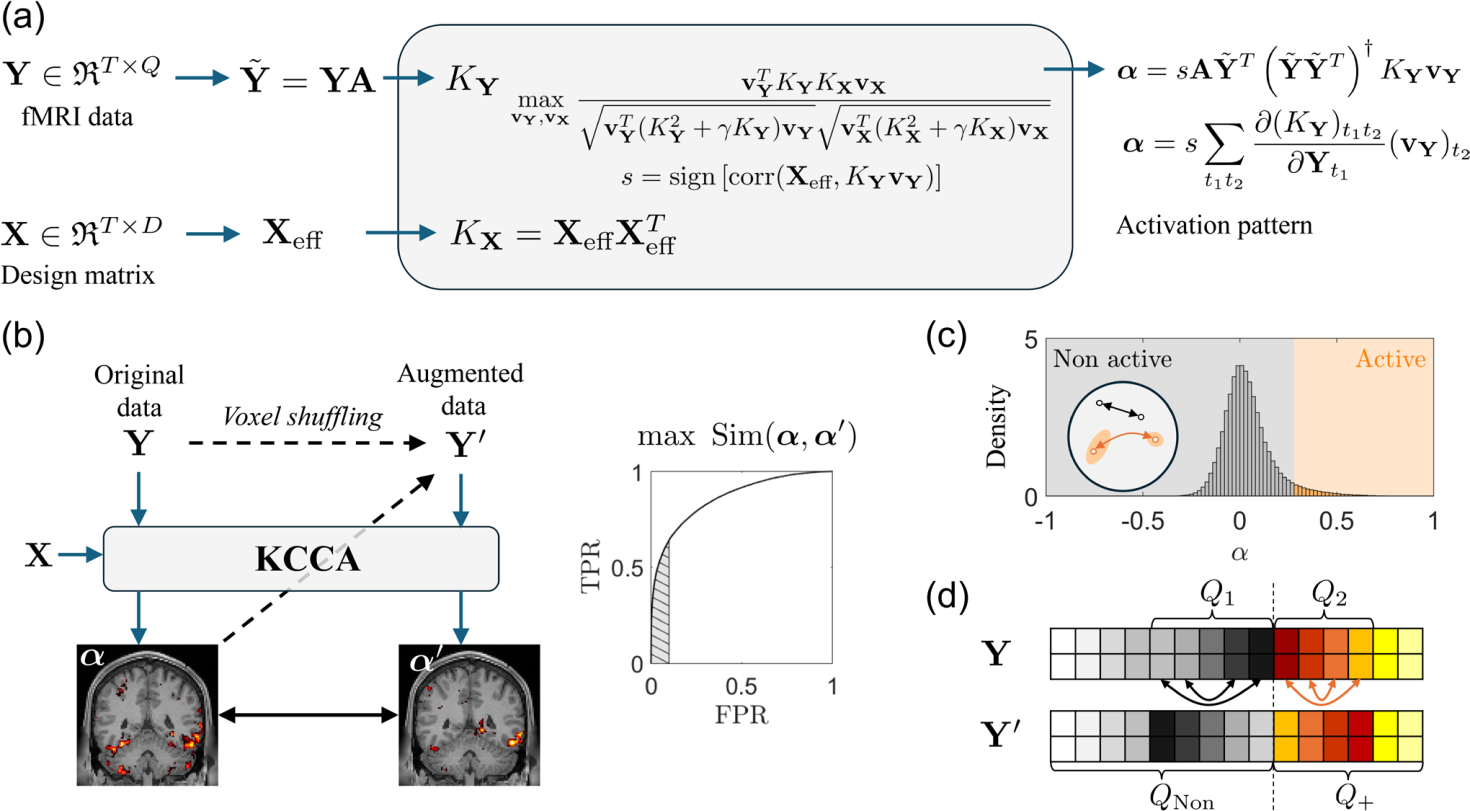
(a) Flowchart for nonlinear kernel-based fMRI activation detection: The fMRI data (upper row) are mapped into kernel space using the kernels listed in Table 3. The design signal (lower row) is mapped into kernel space using a linear kernel. Correlation is computed using KCCA. Once the relationship is established in the kernel space, back-reconstruction methods, as defined in Section 3.6, are applied to extract the activation pattern. (b) Self-supervised learning framework: The original data Y is input into KCCA to obtain the activation map α. After voxel shuffling, the augmented data are labeled as ${\text{Y}}^{\prime }$, corresponding to an activation map $\alpha^{\prime }$ . The ROC curve, shown on the right, is used to measure the similarity between α and $\alpha^{\prime }$ , with similarity quantified as the area under the curve (AUC) for a false positive ratio smaller than 0.1. (c, d) Illustration of voxel shuffling: (c) Distribution of α, computed using the linear kernel from the HCP dataset described in Section 4.1.3. The distributions are similar across different subjects, kernels, and back-reconstruction methods. (d) Visualization of the voxel shuffling process. The top 10% of voxels are labeled as activated. With $Q_{\text{Non}}$ and $Q_{+}$ representing the number of non-activated and activated voxels, respectively, two clusters with voxel counts $Q_{1}$ and $Q_{2}$ are selected near the decision boundary. Shuffling is then performed within each cluster following the method outlined in Section 3.7.

The flow map for the fMRI signal is shown in Figure 2a. Using KCCA, the eigenvectors ${\text{v}}_{\text{X}}$ and ${\text{v}}_{\text{Y}}$ are found to maximize the canonical correlation $r=\text{corr}(K_{\text{X}}{\text{v}}_{\text{X}},$ $K_{\text{Y}}{\text{v}}_{\text{Y}})$ in the feature space. To avoid overfitting, we use

$$r=\underset{{\text{v}}_{\text{X}},{\text{v}}_{\text{Y}}}{\text{max}}\frac{{\text{v}}_{\text{X}}^{T}K_{\text{X}}K_{\text{Y}}{\text{v}}_{\text{Y}}}{\sqrt{{\text{v}}_{\text{X}}^{T}\left(K_{\text{X}}^{2}+\gamma K_{\text{X}}\right){\text{v}}_{\text{X}}}\sqrt{{\text{v}}_{\text{Y}}^{T}\left(K_{\text{Y}}^{2}+\gamma K_{\text{Y}}\right){\text{v}}_{\text{Y}}}},$$ (3)

where γ is a regularization parameter.

### 3.6 Back-reconstruction methods

KCCA maximizes the correlation in the kernel space, but what we want is the activation in the original space $\alpha \in {ℜ}^{Q\times 1}$ . From dimension matching, we assume

$$\text{Y}\alpha \equiv sK_{\text{Y}}{\text{v}}_{\text{Y}},$$ (4)

where $s=\text{sign}\left[\text{corr}\left(X_{\text{eff}},K_{\text{Y}}{\text{v}}_{\text{Y}}\right)\right]$ measures the sign of the correlation between ${\text{X}}_{\text{eff}}$ and the fMRI signal in kernel space.

The intuition behind this assumption is as follows: the correlation in the original space is written as the scalar product 〈Yα|Xβ〉. On the left, the fMRI data are modulated by the activation pattern α and summed over spatial dimensions, while on the right, the design signal is modulated by a contrast-dependent vector β. In the kernel space, this relationship is represented by $\langle K_{\text{Y}}{\text{v}}_{\text{Y}}|K_{\text{X}}{\text{v}}_{\text{X}}\rangle$. By linking the representations in the original and kernel spaces, we directly arrive at Eq. (4). We propose two solutions for the above equation.

First, we insert an identity matrix $\text{I}=\tilde{\text{Y}}\;{\tilde{\text{Y}}}^{T}{\left(\tilde{\text{Y}}\;{\tilde{\text{Y}}}^{T}\right)}^{†}$ on the right-hand side of Eq. (4) and use the relation $\tilde{\text{Y}}=\text{YA}$ . After canceling Y on both sides, we obtain

$$\text{BC }1\left(\text{This study}\right):\alpha =s\text{A}\;{\tilde{\text{Y}}}^{T}{\left(\tilde{\text{Y}}\;{\tilde{\text{Y}}}^{T}\right)}^{†}K_{\text{Y}}{\text{v}}_{\text{Y}}.$$ (5)

The complete derivation of this formula is provided in Appendix A.3.

Second, following the approach of Han et al. (2025), we define ${\text{Y}}_{t}$ as the fMRI data at time t and take the derivation of Eq. (4) with respect to ${\text{Y}}_{t}$. This yields the following expression for α:

$$BC\;2((\text{Han et al., 2025})):\alpha =s\sum_{t_{1}t_{2}}\frac{\partial {\left(K_{Y}\right)}_{t_{1}t_{2}}}{\partial Y_{t_{1}}}{\left(v_{Y}\right)}_{t_{2}},$$ (6)

where the definition of s is consistent with BC 1. The detailed derivation is shown in Appendix A.4. In the previous work (Han et al., 2025), this formulation is referred to as the heat map or sensitivity map, which measures the contribution of each voxel to the signal in kernel space. Here, we treat ${\text{v}}_{\text{Y}}$ as a constant vector so that the back-reconstruction method reduces to the results of the linear kernel in this special case.

For the special case of the linear kernel, as proven in Appendix A.5, both methods reduce to the previously published result up to a constant factor, $\alpha \propto \text{A}{\text{A}}^{T}{\text{Y}}^{T}{\text{v}}_{\text{Y}}$ (Hardoon et al., 2004; Yang et al., 2022). For nonlinear kernels, the two different BC methods will yield different results.

For the activation analysis as shown in Section 4.1, we focus on the new proposed BC 1 method. In the meantime, we compare the two methods under the same condition in Section 4.3. Further discussion for the comparison between the two BC methods is shown in Section 5.2.

### 3.7 The self-supervised learning framework

The self-supervised learning framework uses augmentation methods to generate positive pairs as supervisory examples and then constructs a loss function to maximize their similarity. From the augmentation perspective, this process can be categorized as a one-step or two-step method. The one-step method applies augmentation directly to the data without considering the underlying structure, whereas the two-step method uses the model output to guide data shuffling.

#### 3.7.1 One-step augmentation

The first idea examines the activation robustness under noise injection (Alam, 2014). This method generates augmented examples from the original data with added noise. Specifically, we define ${\text{Y}}^{\prime }=\text{Y}+0.1\in$ , where ∈ is Gaussian white noise. Using ${\text{Y}}^{\prime }$, we compute a new $\alpha^{\prime }$ for both GLM and kernel methods. The distance between α and $\alpha^{\prime }$ is defined as their mean square error (MSE), leading to the formulation of the objective function as follows:

$$\begin{array}{cc} \begin{array}{cc} & \underset{\text{parameters}}{\text{min}}\text{MSE}=∥\alpha -\alpha^{\prime }{∥}^{2}\\ & \text{subject to parameters}\in \text{Table 3}. \end{array} & \end{array}$$ (7)

For practical implementation, first, when computing the loss, both α and $\alpha^{\prime }$ are evaluated using the same kernel with the same parameters, and an additional KCCA is performed to determine $\alpha^{\prime }$ . Second, because the process of generating $\alpha^{\prime }$ involves random variables, statistical averaging is required. Otherwise, the optimization may become trapped in false minima caused by randomness. The results for one-step augmentation are shown in Section 4.2.4. Due to the high randomness, which introduces large statistical variance, this approach incurs a high computational burden for high-dimensional data such as fMRI. Therefore, the one-step augmentation is limited to one-dimensional simulations.

#### 3.7.2 Two-step augmentation

A two-step approach with information-guided augmentation can reduce the randomness introduced during shuffling (Mo et al., 2021). The idea is that isolated activated voxels are rare, so a good method will try to maintain the prediction results even after the voxel location has changed. Specifically, the framework contains data augmentation techniques to shuffle the voxels based on their α value and a way to measure the similarity between the original result and the result after voxel shuffling. The flow map for this method is shown in Figure 2b.

Starting with a specific kernel and given parameters, we use KCCA to obtain the activation pattern α. The distribution of α is shown in Figure 2c. Assume that the top 10% of voxels with large α values are active, and the other 90% are inactive. We denote the number of activated and non-activated voxels as $Q_{+}$ and $Q_{\text{Non}}$ , respectively. Near the decision boundary, we select two clusters: ${\mathbb{Q}}_{1}$ and ${\mathbb{Q}}_{2}$, each with $Q_{1}$ and $Q_{2}$ number of voxels from the non-activated regions and activated regions, respectively, where the blackboard bold name is for the name of the cluster. We then reverse the order within each cluster based on their α values. For instance, for all voxels in ${\mathbb{Q}}_{1}$, we switch the location of the voxel with the largest α with that of the smallest α. The same switches are performed on the voxels with the second largest/smallest α, and so on. This shuffling is repeated for cluster ${\mathbb{Q}}_{2}$. The entire process is depicted in Figure 2d. Note that shuffling is performed before smoothing.

After shuffling, we obtain a new configuration labeled ${\text{Y}}^{\prime }$ and compute the activation pattern $\alpha^{\prime }$ using the same kernel and parameters, and we implement the KCCA again to get a new $v_{Y}^{'}$. By applying different thresholds to $\alpha^{\prime }$ , we define true positive (voxels still activated after shuffling) and false positive (inactive voxels becoming activated after shuffling) to generate a Receiver Operating Characteristic (ROC) curve. An example ROC curve is shown in Figure 2b. This curve, also known as the apparent ROC curve in previous studies (Zhuang et al., 2017), measures shuffling robustness. In prior fMRI neuroscience research, the metric was designed to minimize type 1 errors to reduce the number of false positives (Skudlarski et al., 1999). Similarly, we focus on the voxels that the model strongly predicts as activated, specifically those with relatively large $\alpha^{\prime }$ . We use the Area Under the ROC Curve (AUC) computed with a False Positive Rate (FPR) of less than 0.1 to measure the robustness. We repeat this process to find the best parameters that maximize the AUC.

Even though the two-step augmentation is based on previous outputs and the randomness is reduced, the specific design may not necessarily yield better representations of the data. In other words, activation patterns that exhibit high shuffling robustness may not overlap with gray matter well, particularly for relatively complicated kernel mappings. This problem is later referred to as reduced cross-task performance. Practically, we found that using multiple shuffling methods and averaging the results enhance the robustness of self-supervised learning. Consequently, we implement two shuffling methods. The first shuffling method permutes voxels across the entire region by setting $Q_{1}=Q_{\text{Non}}$ and $Q_{2}=Q_{+}$, while the second shuffling method permutes voxels near the decision boundary by setting $Q_{1}=Q_{2}=0.5Q_{+}$. The AUC values obtained from the two shuffling methods are combined with equal weights. Following the convention of minimizing the objective function, a negative sign is applied to define the final objective.

$$\begin{array}{cc} \begin{array}{cc} & \underset{\text{parameters}}{\text{min}}ℒ=-{\text{AUC}}_{\text{FPR}<0.1}\left(\text{Shu}ffl\text{ing 1}\right)-{\text{AUC}}_{\text{FPR}<0.1}\\ & \;\;\;\;\left(\text{Shu}ffl\text{ing 2}\right),\text{subject to parameters}\in \text{Table 3}. \end{array} & \end{array}$$ (8)

We use the MATLAB Surrogate Optimizer to minimize ℒ (Gutmann, 2001). This optimization is generally a nonlinear problem. Because we employ a surrogate-based derivative-free solver, we adopt a fixed number of optimization steps for each kernel, as summarized in Table 3.

For the bounded linear, hyperbolic tangent, and mixed hyperbolic tangent kernels, we initialize the optimization using the parameters obtained from the linear kernel. For other kernels, we use randomly selected initial conditions as specified in Table 3. Once the optimal parameter combination is identified, we evaluate the activation using the method described in Section 3.8.

The rationale for the self-supervised learning is further validated in Section 4.2. In brief, Section 4.2.1 shows that spatial correlation is preserved after voxel shuffling, while Sections 4.2.2 and 4.2.3 further validate the self-supervised learning assumptions at the subject level. Section 4.2.4 presents results for one-dimensional simulated data with one-step augmentation. Section 4.2.5 compares performance obtained using a single shuffling method—specifically, the first shuffling method, corresponding to including only the first term in Eq. (8)—with performance obtained using both shuffling methods, in which the first and second shuffling methods are combined and both terms in Eq. (8) are incorporated. Section 4.2.6 further evaluates the convergence stability of the proposed surrogate-based optimization. By examining the relative change in the objective function under extended optimization steps, we provide empirical evidence that the selected optimization budgets are sufficient and that the optimization procedure achieves stable solutions across different kernel configurations.

### 3.8 Model validation

We propose five different metrics to characterize the performance of various kernels.

#### 3.8.1 Metric 1: Ground truth

For simulated data described in Section 2.5, Section 2.6 and Section 2.7, where active voxels are known, we generate a ROC curve by setting different thresholds for α. We use the AUC to characterize performance. In fMRI data analysis, minimizing false positives is crucial. The area for the False Positives Ratio (FPR) smaller than 0.1 is commonly used in previous fMRI publications (Skudlarski et al., 1999). We define the final accuracy as the ratio between the kernel method and the baseline model (GLM+GS):

$$r_{\text{Truth}}=\frac{{\text{AUC(kernel)}}_{\text{FPR}<0.1}}{{\text{AUC(GLM+GS)}}_{\text{FPR}<0.1}}.$$ (9)

This ratio is evaluated for two simulated datasets. It does not depend on the number of voxels chosen to be activated. A value $r_{\text{Truth}}>1$ indicates that the kernel method generates better results than GLM+GS, and similarly when $r_{\text{Truth}}<1$ shows worse performance.

#### 3.8.2 Metric 2: Activation in gray matter vs non-gray matter

For task-fMRI data, where the ground truth is unknown, we assume that Blood-Oxygen-Level-Dependent (BOLD) activations occur primarily in gray matter (Logothetis & Wandell, 2004). The rationale includes: first, it has been shown that gray matter has three to four times the cerebral blood flow and volume compared with white matter (Gawryluk et al., 2014). Second, the BOLD signal in gray matter is three to six times larger than in white matter, with the latter also showing time delays or even negative correlations with stimulation (Schilling et al., 2023). The standard choice for HRF may not be suitable for white matter; as a result, this increases the rationale for treating white matter as false activation. Third, unlike surface-based methods, which require anatomical structures (Brodoehl et al., 2020; Mejia et al., 2020), or task denoising methods (Yang et al., 2020), our volume-based approach does not require such information, making gray matter suitable for independent testing. Fourth, this metric is consistent with earlier investigations that examined whether identified activations align with detailed gray matter profiles (Han et al., 2025; Yang et al., 2018, 2025).

Following the same idea in Metric 1, we define true positive activation in gray matter and false positive activation not in gray matter to generate the ROC curve. Gray matter is generated from the SPM package segmentation with a probability threshold set as greater than 0.5. One of the examples is shown in Figure 1b. Note that the number of voxels in gray matter is significantly larger than the number of activated voxels, which results in the AUC being close to 0.5.

Similarly, we aim to minimize false positives and reduce activation in non-gray matter, particularly in voxels that the method strongly predicts as active. We define the objective ratio for gray matter activation as follows:

$$r_{\text{Gray}}=\frac{{\text{AUC(kernel)}}_{\text{FPR}<0.1}}{{\text{AUC(GLM+GS)}}_{\text{FPR}<0.1}}.$$ (10)

This ratio, $r_{\text{Gray}}$ , is evaluated for the HCP dataset and in-house scans. A large ratio indicates that the activation is more concentrated in gray matter than in other areas. This ratio does not depend on the activation threshold but does depend on the threshold for gray matter. However, we examined the output probabilities from segmentation using the SPM package, and they are concentrated around 1 or 0, leaving a few voxels on the margin.

#### 3.8.3 Metric 3: Shuffling robustness

Similar to training accuracy in traditional machine learning, we introduce a factor to characterize shuffling robustness. As a note, we can also compute the shuffling based on GLM+GS, even if it does not have any parameters. We use ℒ defined in Eq. (8) to characterize the relative accuracy between kernel and baseline model

$$r_{\text{Shu}ffl\text{ing}}=\frac{ℒ\left(\text{kernel}\right)}{ℒ\left(\text{GLM+GS}\right)}.$$ (11)

This metric measures the robustness of certain activation patterns under voxel shuffling. It serves two purposes: characterizing our self-supervised learning method and providing an additional cross-validation of our results, especially for task-fMRI when the ground truth is not available.

#### 3.8.4 Metric 4: Activations in regions of interest

While activation in gray matter is generally desired, as BOLD signal strength is typically higher there, it is not sufficiently specific for different tasks. This section introduces a more task-related mask to further characterize model performance.

For the working memory task, previous studies on the HCP and other working memory task-fMRI focus on activation in the left prefrontal cortex region with MNI coordinates (−44, 14, 29) (Cole et al., 2012; Lamichhane et al., 2020). The regions-of-interest (ROI) mask $M_{\text{ROI}}$ is then defined as a spherical region with a 6 mm radius centered at this location, which also covers the left inferior frontal gyrus pars opercularis at (−46, 10, 26) (Rottschy et al., 2012). The mask is shown in Figure 1c.

For the episodic memory task, previous publications usually focus on hippocampal activation (Jin et al., 2012). The hippocampus plays a central role in episodic memory encoding and retrieval, particularly through its well-established functions in associative binding, novelty detection, and pattern separation. During encoding, hippocampal circuits integrate perceptual and contextual features into conjunctive memory traces, while during recognition, they support the reinstatement and discrimination of stored representations from similar inputs. The centers of the hippocampus are defined using MNI coordinates (24,−12,−20) and (−24,−12,−20) (Tzourio-Mazoyer et al., 2002). For each center, we define the mask by placing a sphere with a 6 mm radius. The ROI mask $M_{\text{ROI}}$ is shown in Figure 1d.

Because this region is very small, we do not use the ROC analysis proposed previously and instead compute a direct summation of the activation within this region (Lamichhane et al., 2020)

$$r_{\text{ROI}}=\sum M_{\text{ROI}}\bar{\alpha },$$ (12)

where $\bar{\alpha }$ is α normalized by the standard deviation, and $M_{\text{ROI}}$ is the ROI mask.

#### 3.8.5 Metric 5: Eigendecomposition

Previous analyses focused on final performance rather than the kernel itself. To characterize the information encoded in the kernel matrix, we note that all kernels listed in Table 3 are symmetric and, therefore, admit a valid eigendecomposition $K_{\text{Y}}=\sum_{i}\lambda_{i}\nu_{i}\nu_{i}^{T}$, where $\lambda_{i}$ is the eigenvalue and $\nu_{i}\in {ℜ}^{T\times 1}$ is the corresponding eigenvector. Each eigenvector $\nu_{i}$ defines a direction in the implicit feature space that captures a distinct mode of data variation, with its importance measured by $\lambda_{i}$ (Shawe-Taylor & Cristianini, 2004). In the principal component analysis problem, eigenvectors corresponding to larger eigenvalues are selected as features along the dominant directions. In real fMRI data, due to the high noise level, we find that selecting all eigenvectors, rather than examining only the $\nu_{i}$ associated with the largest eigenvalues, provides more stable performance across all kernels. To characterize the similarity between $\nu_{i}$ and ${\text{X}}_{\text{eff}}$ , we define

$$r_{\text{Corr}}=\underset{\text{parameters},i}{\text{max}}\left|\text{corr}\left(\nu_{i},{\text{X}}_{\text{eff}}\right)\right|.$$ (13)

We propose this metric for several reasons. First, for a linear problem without noise, matrix ${\text{K}}_{\text{Y}}$ is rank 1 with $\nu_{i}={\text{X}}_{\text{eff}}$ , yielding $r_{\text{Corr}}=1$ . Second, for a linear problem with noise or for a nonlinear kernel, a rigorous formulation is difficult to derive. A large $r_{\text{Corr}}$ can indicate a high overlap with ${\text{X}}_{\text{eff}}$ . Although a rigorous connection to KCCA is implicit, a large value still indicates high flexibility of the kernel mapping. Third, we use the absolute value because the eigenvalue problem in KCCA contains squared terms for $K_{\text{Y}}$ (Yang et al., 2018). As a result, sign differences do not affect the outcome.

Based on these observations, we assume that a high $r_{\text{Corr}}$ computed with a given kernel for a specific subject indicates that the kernel encodes more relevant information, reflecting this property primarily through the kernel function itself. Practically, for a nonlinear kernel with unknown parameters listed in Table 3, we implement a grid search to find $r_{\text{Corr}}$ across all possible mappings. As a result, it is expected that a more complicated kernel, such as the mixed hyperbolic tangent, yields a higher $r_{\text{Corr}}$ than the hyperbolic tangent kernel. As noted, the grid search is not implemented in γ, as that is the regularization parameter for KCCA.

### 3.9 Statistical test

All evaluation metrics have the following properties: first, each metric yields a scalar value for each subject, with larger values indicating better performance (e.g., higher normalized activation in gray matter or ROIs). Second, the metric is normalized at the subject level, making cross-subject comparisons valid. Given two vectors of such performance scores obtained from different methods, the t-test evaluates whether the observed difference in their averages is unlikely to have occurred by chance. The null hypothesis is that the first set does not perform better than the second set. Rejecting this null hypothesis indicates statistically significant evidence that the first method achieves higher performance.

For all statistical tests listed in the study, statistical significance between the two methods was evaluated using a paired t-test across subjects. Since the analysis involved a single hypothesis test comparing the average performance of the two methods, correction for multiple comparisons was not required.

### 3.10 Activation threshold

Since the activation represents continuous values, we need criteria to establish the activation threshold. A commonly used approach is to resample resting-state fMRI data to compute the correlation and treat this correlation as the null distribution to define the FPR (Bullmore et al., 2001; Laird et al., 2004; R. R. Nandy & Cordes, 2003).

However, due to kernel mapping, tracking the exact values during the computation pipeline is challenging, especially when the method involves several normalization steps. In this study, we compute the null distribution by shuffling ${\text{v}}_{\text{Y}}$. Specifically, we apply random time shuffling to ${\left({\text{v}}_{\text{Y}}\right)}_{\text{null}}$ and then use the randomly shuffled ${\left({\text{v}}_{\text{Y}}\right)}_{\text{null}}$ as a constant to compute $\alpha_{\text{null}}$ . The threshold is then gradually decreased until the false activation rate reaches 0.05, analogous to the commonly used p value in previous publications. For GLM+GS, we randomly shuffle ${\text{X}}_{\text{eff}}$ to compute $\alpha_{\text{null}}$ .

Compared with the previous studies, this study exhibits a relatively high FPR, because we have not changed the spatial domain, with shuffling performed only in the kernel dimension. Consequently, the computed $\alpha_{\text{null}}$ may yield relatively high values, necessitating a higher FPR. Nevertheless, the specific threshold value does not affect the accuracy.

## 4 Results

### 4.1 Activation analysis for task-fMRI

#### 4.1.1 fMRI simulation 1

**Fig. 3.**
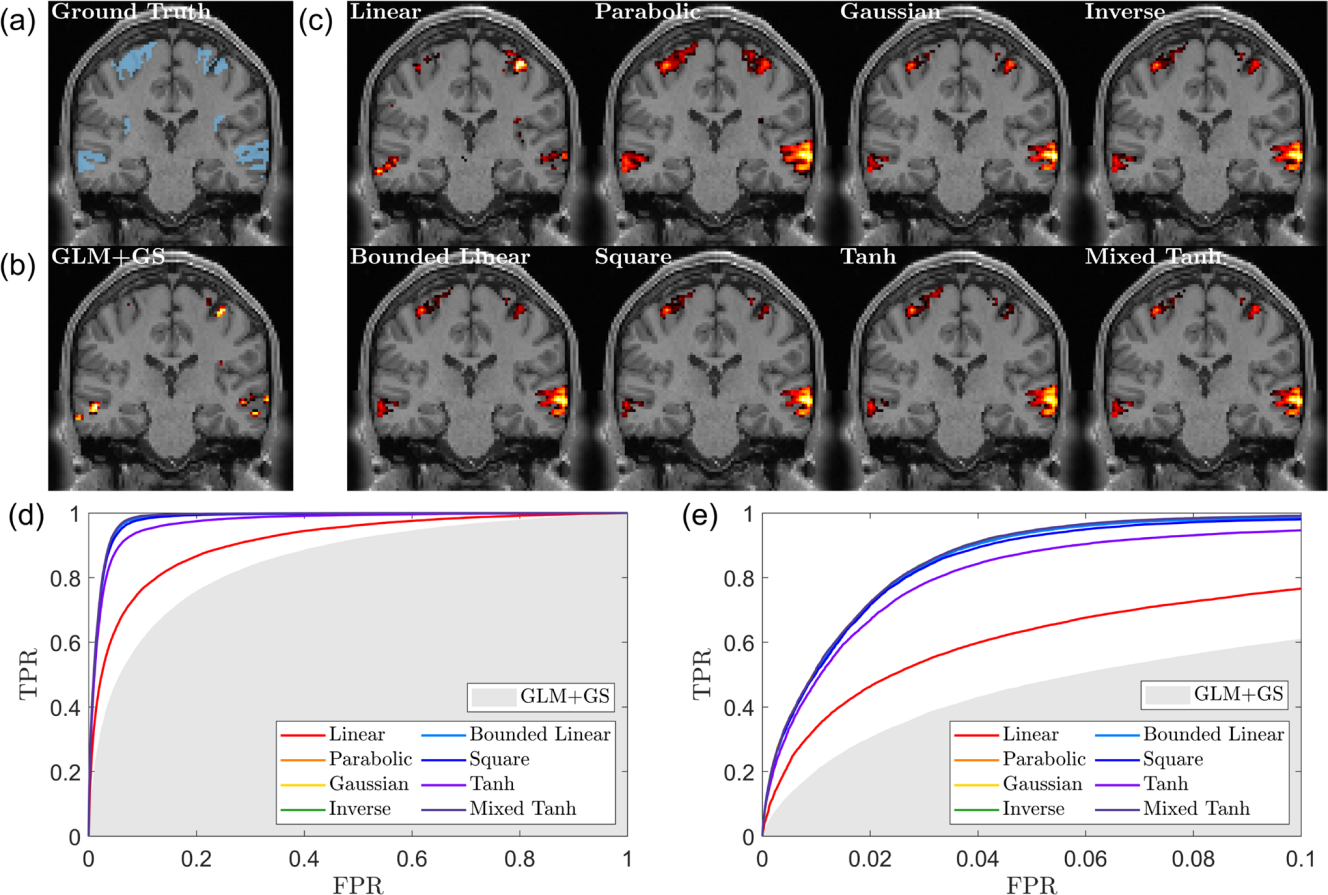
Ground truth, activation map, and ROC curve for the first simulated fMRI data. (a) The ground truth, with the activation area highlighted in blue, in the T1 image at the corresponding location for reference. (b) Result from GLM+GS. The color indicates the voxel with p<0.05 , with a brighter color (yellow) for the larger α value. (c) Activation pattern for eight different kernels using KCCA. (d) ROC curve for different kernels. The gray shading area indicates the AUC obtained from GLM+GS. (e) A focused view of (d) for FPR <0.1 . Nonlinear kernels generate better performance than the linear kernel.

Figure 3 shows an example of the first simulation. The activation threshold is given using p<0.05 . The ground-truth activated regions in Figure 3a (blue) consist of activations in both the mid-temporal gyrus (lower) and the precentral gyrus (upper). The activation pattern detected using GLM+GS is relatively small, and the linear kernel slightly improves the results but not by much. However, for all the nonlinear kernels, two clusters can be clearly seen with fine detail.

In Figure 3d and e, we present the AUC values for all the different kernels, with the gray-shaded area indicating the range for GLM+GS. For this subject, all kernels outperform GLM+GS by a relatively large margin. Specifically, for AUC with FPR<0.1 , GLM+GS achieves a value of 0.0432, which is selected between 0.035 and 0.05 to maintain consistent signal strength in the same region. The linear kernel achieves an AUC of 0.0587, while the hyperbolic tangent kernel reaches 0.0792. The other six kernels yield similar values, ranging between 0.0840 and 0.0845, indicating that these kernels provide stable and improved performance compared with GLM+GS.

**Fig. 4.**
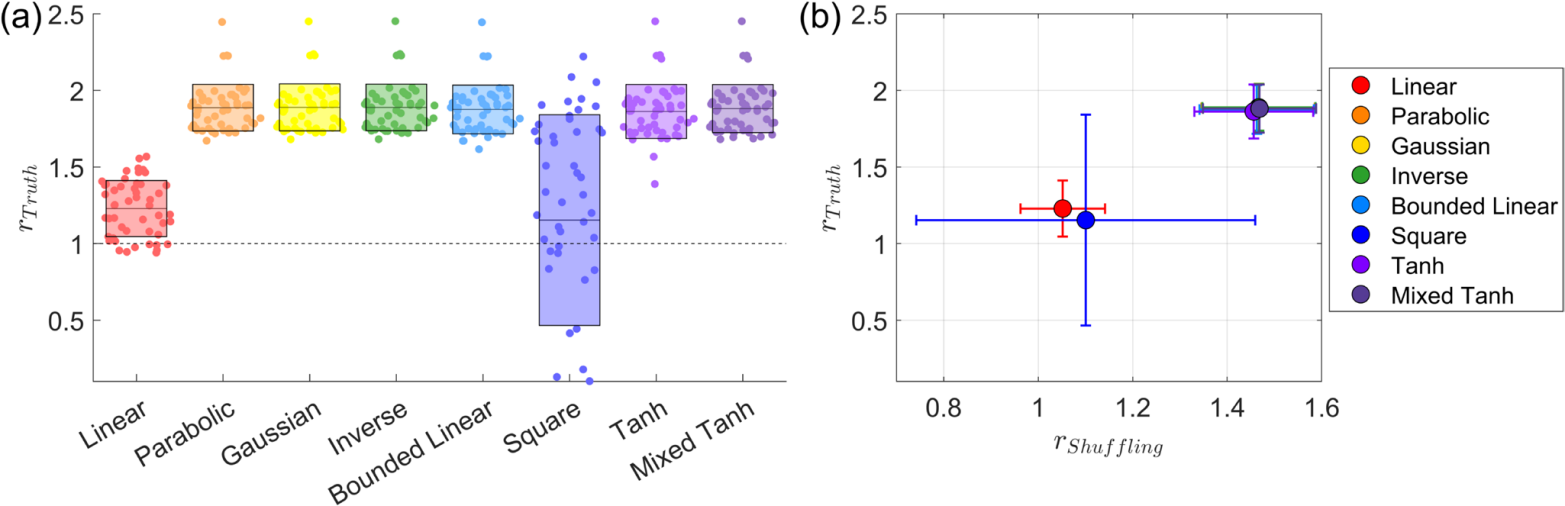
Subject-wise average performance on the first simulated fMRI dataset. (a) Swarm plot of the AUC ratio evaluated using the ground truth. Results are based on 50 subjects from the first simulated dataset. Each dot represents one subject. The horizontal line and rectangle bar indicate each method’s mean and variance. (b) Relationship between shuffling robustness $r_{\text{Shu}ffl\text{ing}}$ and accuracy $r_{\text{Truth}}$ for different kernels. Each dot represents the average over 50 subjects. In general, activations with greater robustness to shuffling also exhibit higher accuracy. Except for the square kernel, nonlinear kernels outperform linear kernels in both robustness and accuracy.

Figure 4 presents the subject-wise average for this simulated dataset. In Figure 4a, the swarm plot displays the performance of all eight kernels, with exact values provided in Table 5. The linear kernel shows a performance increase of approximately 20%. In contrast, all other kernels—except the square kernel—achieve performance improvements of more than 80%. The square kernel performs poorly, as indicated by its large variance. Our empirical analysis suggests that, for this problem, the signal is added in a relatively uniform manner. As shown in Table 2, the signal strength is relatively low, yet the method still achieves a decent AUC value, indicating that the problem is relatively easy to learn but still requires a proper mapping function. Neither the linear nor the square kernels have parameters to tune for the mapping; their fixed mappings prevent them from adapting effectively. In contrast, the other kernels can capture the underlying structure more effectively and achieve relatively high accuracy. Another way to interpret this result is that nonlinear kernels can effectively identify optimal parameters that exhibit strong shuffling robustness. As shown in Figure 13a, with proper nonlinear kernel mapping, most subjects reach −ℒ values, defined in Eq. (8), exceeding 0.18, which is close to the maximum value of 0.2 and is the highest among all datasets. Consequently, the large difference in shuffling robustness between linear and nonlinear kernels also leads to a substantial difference in accuracy.

Figure 4b presents the subject-wise average result for shuffling robustness and accuracy. We observe that the linear kernel exhibits the lowest shuffling robustness, and its average accuracy is also relatively low. In contrast, all nonlinear kernels, except for the square kernel, achieve both high accuracy and strong shuffling robustness. This result supports our assumption that the self-supervised learning approach can effectively identify the optimal parameters for nonlinear kernel mapping, ensuring both robustness and accuracy in activation detection.

#### 4.1.2 fMRI simulation 2

**Fig. 5.**
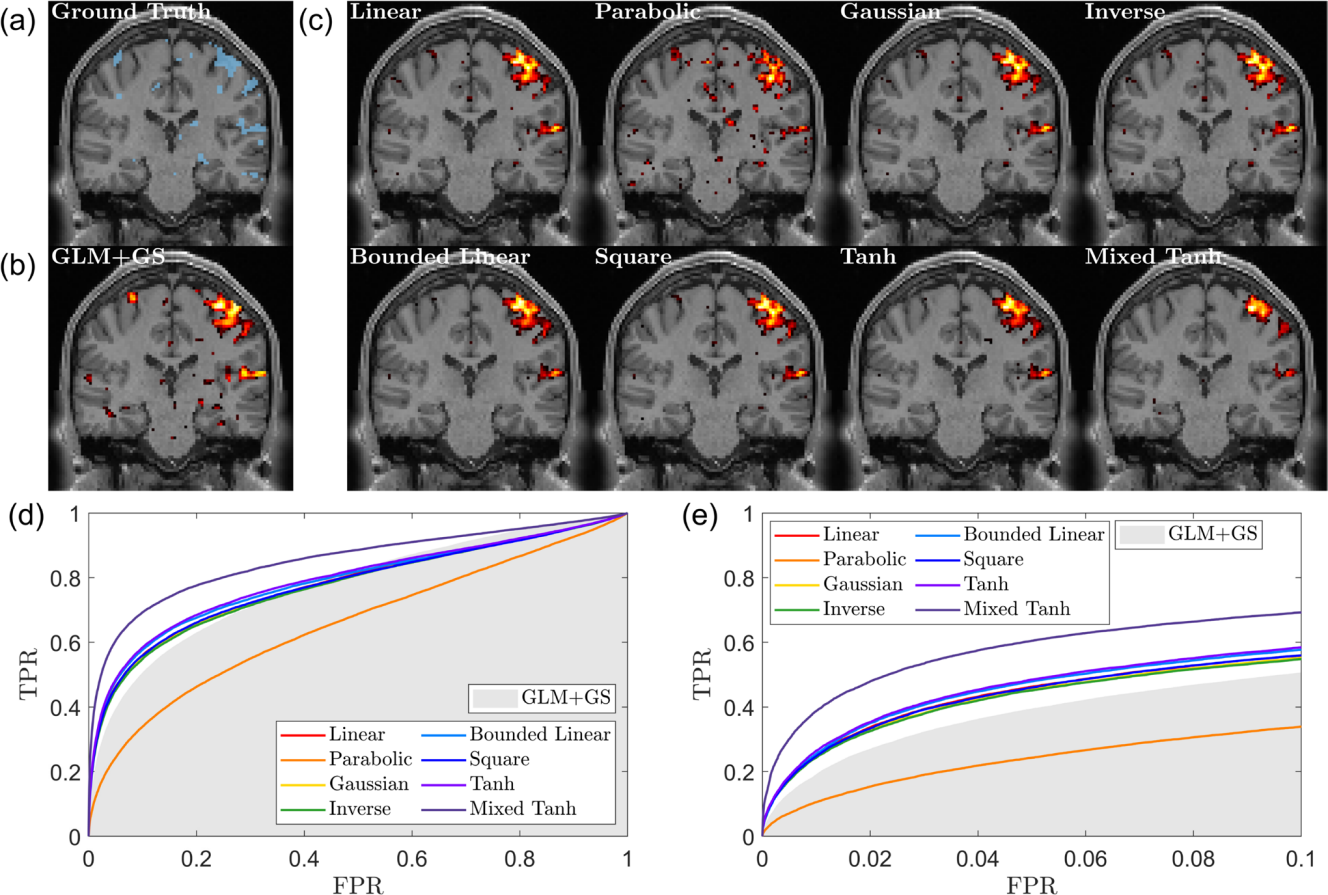
Ground truth, activation map, and ROC curve for the second simulated fMRI data. (a) The ground truth, with the activation area highlighted in blue, in the T1 image at the corresponding location for reference. (b) Result from GLM+GS. The color indicates the voxel with p<0.05 , with a brighter color (yellow) for the larger α value. (c) Activation pattern for eight different kernels using KCCA. (d) ROC curve for different kernels. The gray shading area indicates the AUC obtained from GLM+GS. (e) A focused view of (d) for FPR <0.1 . The mixed hyperbolic tangent kernel demonstrates the highest performance, achieving the largest AUC.

Figure 5a presents an example from 1 of the 20 simulated subjects from the second simulated dataset. In this case, the ground-truth activation consists of relatively small clusters compared with the first simulation shown in this study. Figure 5b and c compares the performance of GLM+GS with eight different kernels. Except for the parabolic kernel, all other kernels produce activation patterns that closely resemble those of GLM+GS. The main difference is that most kernels generate relatively larger activation clusters than the ground truth. In contrast, the mixed hyperbolic tangent kernel, under the same thresholds method, produces smaller cluster sizes, effectively reducing false activations while maintaining accurate detection.

In Figure 5c and d, we present the AUC values with FPR<0.1 for all methods. The baseline GLM+GS achieves an AUC of 0.0366, while the linear kernel improves slightly to 0.0428. The best-performing method for this subject is the mixed hyperbolic tangent kernel, which achieves an AUC of 0.0564. In contrast, the worst-performing method is the parabolic kernel, with an AUC of 0.0228. The other kernels fall within a narrow range between 0.0417 and 0.0447.

**Fig. 6.**
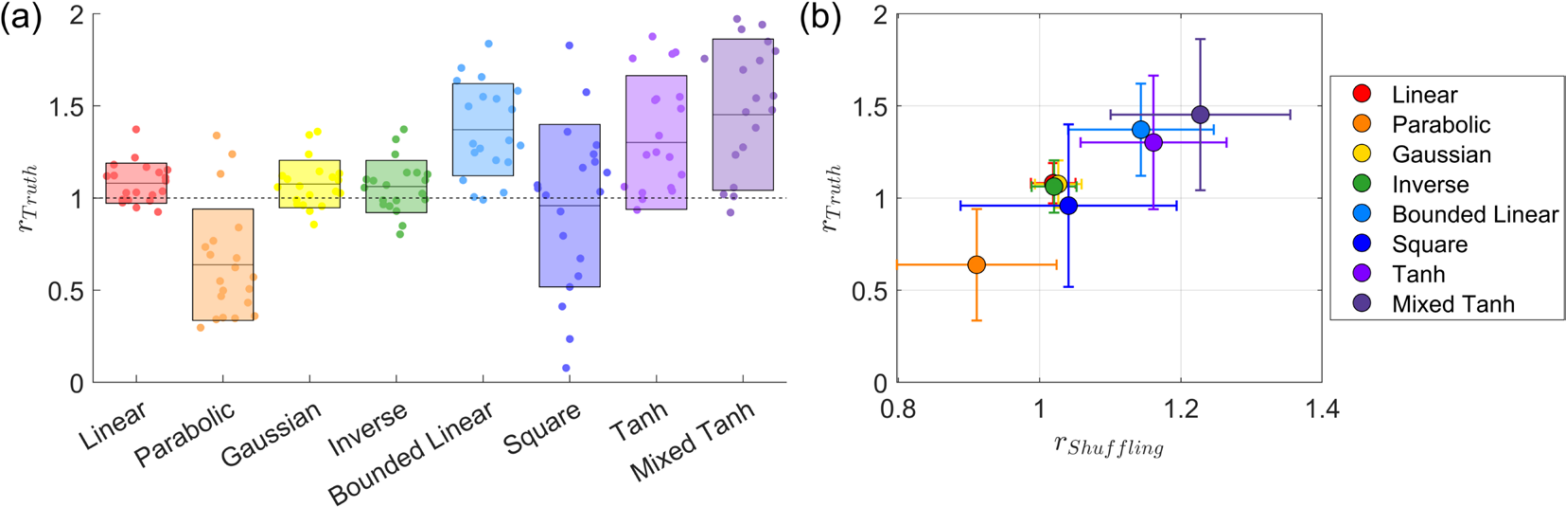
Subject-wise average performance on the second simulated fMRI dataset. (a) Swarm plot for the AUC ratio evaluated using ground truth. Results based on 20 subjects taken from the second simulated fMRI dataset. Each dot represents the result from one subject. The horizontal line and the rectangle bar indicate each method’s mean and variance. (b) Relationship between shuffling robustness $r_{\text{Shu}ffl\text{ing}}$ and accuracy $r_{\text{Truth}}$ for different kernels. Each dot represents the average over 20 subjects. In general, activation with robust shuffling also has higher accuracy. The mixed hyperbolic tangent has the maximum performance for both shuffling and accuracy.

Figure 6 presents the subject-wise average for the second simulated fMRI data. In Figure 6a, the swarm plot illustrates the performance distribution across different kernels. Compared with the first simulation, the performance differences between kernels are more pronounced. Specifically, the linear kernel exhibits very small variance, indicating highly stable performance that closely tracks the baseline model. The Gaussian and inverse kernels show nearly identical behavior to the linear kernel. Similar to the first simulation, the square kernel performs poorly, exhibiting substantially larger variance, with many subjects falling below the baseline.

Figure 6b presents the subject-wise average ratio between shuffling robustness and accuracy. A strong linear relationship is observed between these two metrics. The mixed hyperbolic tangent kernel achieves the highest performance in both shuffling robustness and accuracy, whereas the parabolic kernel performs the worst in both cases.

#### 4.1.3 HCP dataset

**Fig. 7.**
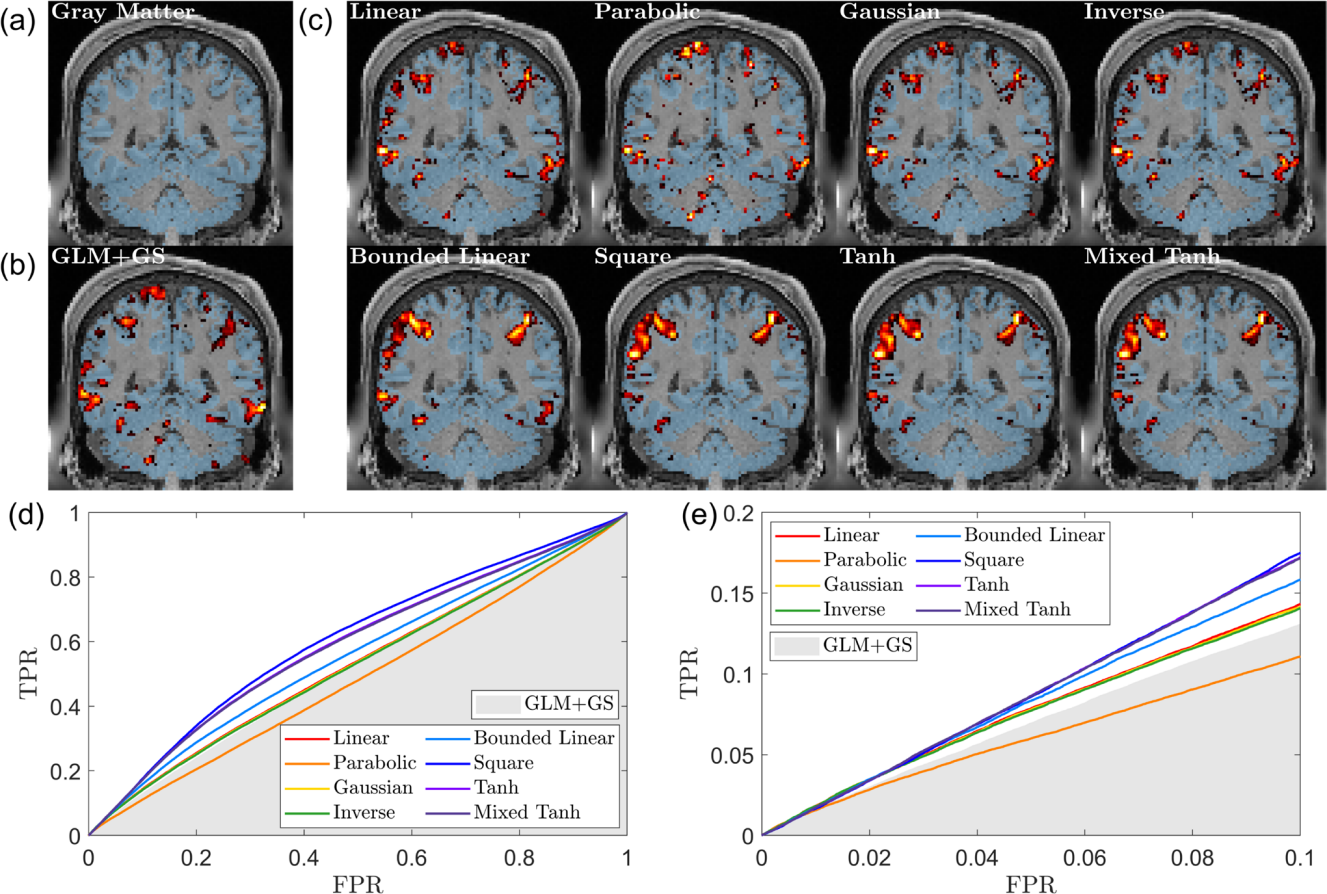
Gray matter, activation map, and ROC curve for task-fMRI data (HCP dataset). (a) The gray matter (shown in blue) was determined using segmentation probabilities larger than 0.5 , overlaid on the T1-weighted image. (b) Activation map obtained using GLM+GS. Voxels with p<0.05 are color coded, where brighter colors (yellow) indicate larger α values; the gray matter mask (blue) and T1-weighted image are overlaid for reference. (c) Activation pattern for eight different kernels using KCCA. (d) ROC curves for the different kernels, with the gray shading area representing the AUC obtained from GLM+GS. (e) A focused view of (d) for FPR <0.1 . The parabolic kernel demonstrates poor performance, as indicated by scattered activations and the corresponding curve. In contrast, the bounded kernel such as tanh or mixed tanh demonstrates optimal performance in detecting activation in gray matter.

Figure 7 presents one of the selected examples, with Figure 7a showing that the gray matter serves as the reference. The results from GLM+GS are shown in Figure 7b. Results for the eight kernels resulting from the back-reconstruction methods are displayed in Figure 7c.

The activation patterns obtained from the eight kernels are approximately categorized into three groups. The first group consists of the parabolic kernel, which produces many small clusters within the brain, indicating an unreliable activation pattern. The linear, Gaussian, and inverse kernels exhibit structures that closely resemble the baseline GLM+GS model, with the primary difference being that the baseline model appears more smoothed. The bounded linear, square, hyperbolic tangent, and mixed hyperbolic tangent kernels share a similar activation structure.

Figure 7d and e displays ROC curves for the different kernels, using the gray matter as the ground truth. Since most voxels in the gray matter are inactive, the AUC values are close to 0.5. Compared with GLM+GS, indicated by the gray-shaded area, the mixed hyperbolic tangent kernel achieves the highest AUC and identifies a larger number of activated voxels within the gray matter. In contrast, the parabolic kernel shows poor performance, with its curves within the gray-shaded area. Comparing the exact AUC values for FPR <0.1 , GLM+GS achieves $6.89\times 10^{-3}$ , while the linear kernel reaches $7.62\times 10^{-3}$ . Among nonlinear kernels, the worst performer is the parabolic kernel at $5.91\times 10^{-3}$ , whereas the best results are obtained with the square and hyperbolic tangent kernels, both at $8.63\times 10^{-3}$ . The mixed hyperbolic tangent kernel performs slightly lower at $8.61\times 10^{-3}$ . Overall, these results suggest that the square, hyperbolic tangent, and mixed hyperbolic tangent kernels outperform the others, the parabolic kernel performs the worst, and the Gaussian and inverse kernels provide no significant improvement over the linear kernel.

**Fig. 8.**
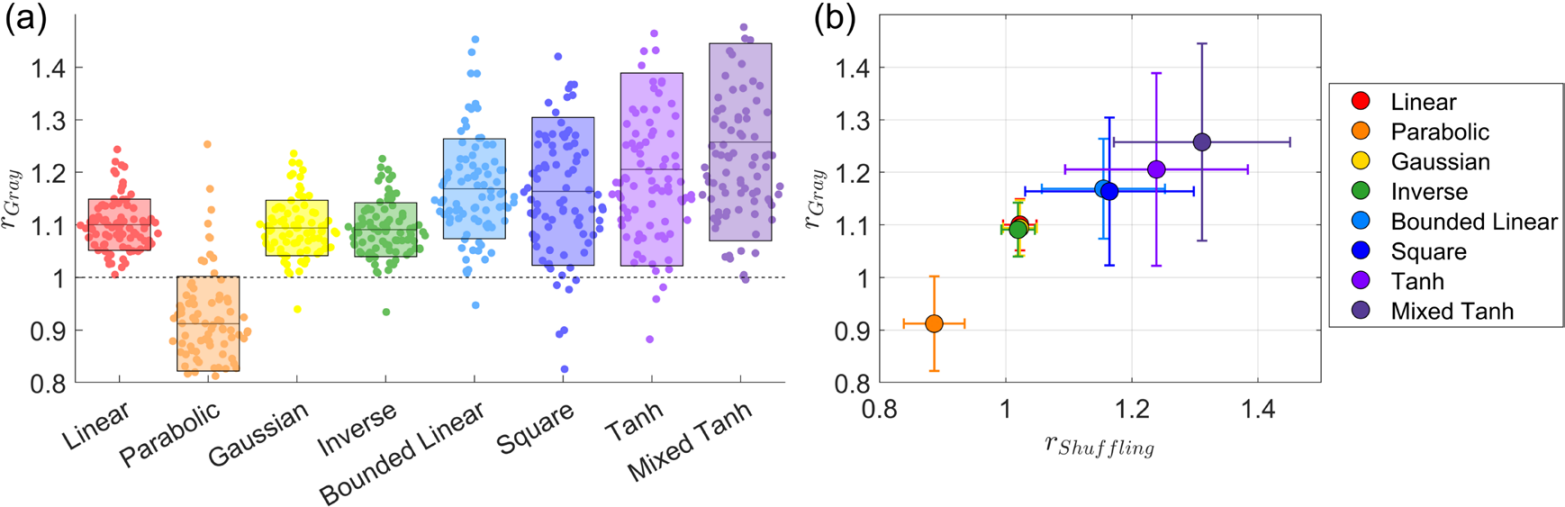
Subject-wise average performance on the HCP dataset. (a) Swarm plot for the AUC ratio evaluated using ground truth. Results based on 87 subjects taken from the HCP dataset. Each dot represents the result from one subject. The horizontal line and the rectangle bar indicate each method’s mean and variance. (b) Relationship between shuffling robustness $r_{\text{Shu}ffl\text{ing}}$ and accuracy $r_{\text{Gray}}$ for different kernels. In general, activation with robust shuffling also has higher accuracy. The mixed hyperbolic tangent has the maximum performance for both shuffling and accuracy.

In Figure 8a, we present the subject-wise average results for the HCP dataset, where $r_{\text{Gray}}$ measures the relative gray matter overlap using GLM+GS as a reference. On average, the mixed hyperbolic tangent method performs the best, showing a 25.74% increase compared with GLM+GS. In contrast, the linear kernel exhibits a 10.03% increase, indicating that our nonlinear kernels can effectively avoid activations in undesired regions compared with traditional methods. Conversely, the parabolic kernel performs the worst for the HCP dataset, with a ratio of -8.79%, indicating less gray matter overlap compared with GLM+GS. To further validate this result, in the first column of Table 4, we list the statistical tests between the mixed hyperbolic tangent kernel and the other kernels. For the HCP dataset, the mixed hyperbolic tangent kernel significantly outperforms other kernels.

**Table 4.** Statistical tests for gray matter overlap with activation for task-based fMRI.

Kernel name	HCP dataset	In-house scans
Linear	<0.001	0.009
Parabolic	<0.001	0.018
Gaussian	<0.001	0.042
Inverse	<0.001	0.003
Bounded linear	<0.001	0.001
Square	<0.001	0.024
Tanh	<0.001	0.033

One-sided paired t-test p-values comparing the gray-matter overlap metric $r_{\text{Gray}}$ defined in Eq. (10), between the mixed hyperbolic tangent kernel and all other kernels.

In Figure 8b, we observe a strong linear relationship between $r_{\text{Shu}ffl\text{ing}}$ and $r_{\text{Gray}}$ , with a correlation coefficient of 0.96. This indicates that activations with high robustness to the shuffling also tend to prefer gray matter. This not only validates our assumption that self-supervised learning based on voxel shuffling is suitable for fMRI activation detection but also provides evidence using different metrics to characterize activation patterns even without knowing the ground truth.

#### 4.1.4 In-house scans

**Fig. 9.**
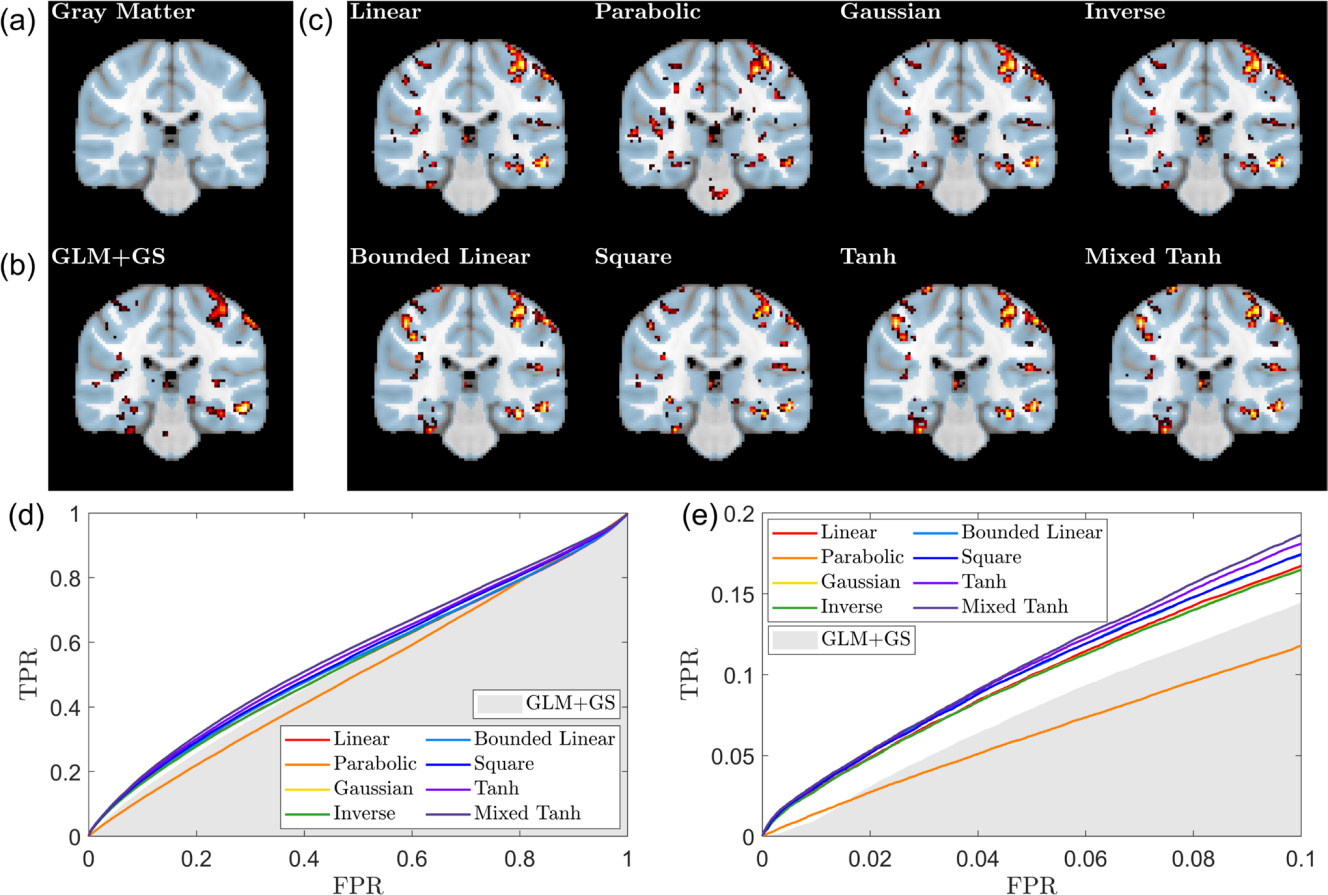
Gray matter, activation map, and ROC curve for task-fMRI data (In-house scans). (a) The gray matter (shown in blue) was determined using segmentation probabilities larger than 0.5 , overlaid on the T1-weighted image. (b) Activation map obtained using GLM+GS. Voxels with p<0.05 are color coded, where brighter colors (yellow) indicate larger α values; the gray matter mask (blue) and T1-weighted image are overlaid for reference. (c) Activation pattern for eight different kernels using KCCA. (d) ROC curves for the different kernels, with the gray shading area representing the AUC obtained from GLM+GS. (e) A focused view of (d) for FPR <0.1 . GLM+GS and the parabolic kernel show some activations in the brain stem. In contrast, the mixed hyperbolic tangent kernel demonstrates optimal performance in detecting activation in gray matter.

We utilized in-house scans providing another task-fMRI dataset to evaluate the performance of nonlinear kernel methods. An example from one subject performing the faces task with the EC contrast is shown in Figure 9. For the activation pattern, Figure 9b presents the results from GLM+GS as a reference, showing some activations in the brain stem, which are likely related to cardiac pulsation and not memory activations. In contrast, Figure 9c shows that, except for the parabolic kernel, brain stem activations disappear when using kernel methods. Another notable effect is that hippocampus activations become stronger for the hyperbolic tangent and mixed hyperbolic tangent kernels.

Figure 9d and e presents the AUC values measured by activation overlays on the gray matter for this subject. As a reference, GLM+GS achieves a value of $0.75\times 10^{-2}$ , while the linear kernel reaches $0.96\times 10^{-2}$ . The top two performing kernels are the mixed hyperbolic tangent and hyperbolic tangent, with values of $1.05\times 10^{-2}$ and $1.03\times 10^{-2}$ , respectively. The bounded linear and square kernels perform similarly, both achieving $1.00\times 10^{-2}$ . The Gaussian and inverse kernels also show similar performance to the linear kernel, both with values of $0.94\times 10^{-3}$ . The parabolic kernel performs the worst, with a value of $0.61\times 10^{-2}$ .

**Fig. 10.**
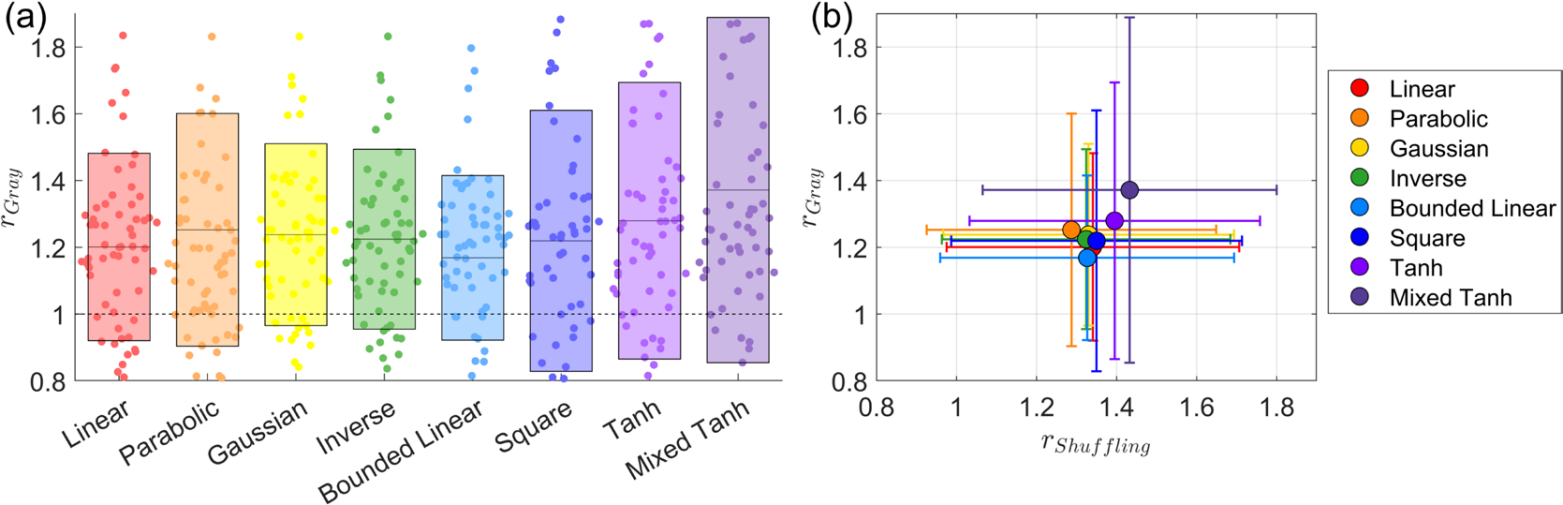
Subject-wise average performance on the In-house scans. (a) Swarm plot for the AUC ratio evaluated using ground truth for the in-house scans. Each dot represents the result from one case. The horizontal line and the rectangle bar indicate each method’s mean and variance. (b) Relationship between shuffling robustness $r_{\text{Shu}ffl\text{ing}}$ and accuracy $r_{\text{Gray}}$ for different kernels. In general, activation with robust shuffling also has higher accuracy. The mixed hyperbolic tangent has the maximum performance for both shuffling and accuracy.

Figure 10 presents the subject-wise average for all 16 subjects across 2 tasks and 2 contrasts. In Figure 10a, we show a swarm plot of the averages across all 64 cases., along with the mean and variance for each kernel method. The specific improvement ratios for AUC with FPR <0.1 are reported in Table 5. This dataset exhibits relatively larger variance than HCP dataset. Several factors may contribute to this variance. First, we observe that GLM+GS does not perform well, usually because these subjects involve relatively large head movements or because low signal strength leads to a higher proportion of voxels exhibiting negative correlation with the effective design signal, which causes the baseline model to have relatively low gray matter overlap with activations. Further validation of this result is shown in Figure 13d for the non-normalized values. Second, part of the variance comes from the metric definition, with the baseline used as the denominator. An alternative definition is to use subtraction between the kernel and the baseline to reduce variance. However, the ratio provides a clearer interpretation of improvement, whereas subtraction only yields an area difference that does not serve a practical meaning.

**Table 5.** Comparison of different back-reconstruction methods.

	fMRI simulation 1		fMRI simulation 2	
Kernel name	BC 1 (This study)	BC 2 (Han et al., 2025)	BC 1 (This study)	BC 2 (Han et al., 2025)
Linear	22.86%	22.86%	8.00%	8.00%
Parabolic	88.69%	9.65%	−36.19%	13.40%
Gaussian	88.91%	12.66%	7.56%	$\underline{9.94\text{\%}}$
Inverse	$\underline{88.86\%}$	12.34%	6.22%	8.72%
Bounded linear	87.54%	11.52%	$\underline{37.04\text{\%}}$	8.31%
Square	15.32%	−6.20%	−4.16%	2.98%
Tanh	86.20%	$\underline{58.64\%}$	30.11%	−16.29%
Mixed tanh	88.10%	66.24%	45.19%	6.20%
	HCP dataset		In-house scans	
Kernel name	BC 1 (This study)	BC 2 (Han et al., 2025)	BC 1 (This study)	BC 2 (Han et al., 2025)
Linear	10.03%	10.03%	20.10%	20.10%
Parabolic	−8.79%	10.01%	25.23%	20.32%
Gaussian	9.41%	12.68%	23.79%	22.14%
Inverse	9.09%	10.12%	22.42%	22.35%
Bounded linear	16.87%	10.26%	16.85%	$\underline{30.85\text{\%}}$
Square	16.37%	$\underline{16.96\text{\%}}$	21.93%	22.84%
Tanh	$\underline{20.55\text{\%}}$	11.27%	$\underline{27.95\text{\%}}$	23.41%
Mixed tanh	25.74%	24.27%	37.14%	31.14%

The performance improvement ratios for the partial ROC with FPR <0.1 are reported. For each dataset and each back-reconstruction method, the best-performing method is shown in **bold**, and the second-best method is shown as underlined.

To further validate the results, in the second column of Table 5, we list the statistical tests between the mixed hyperbolic tangent kernel and the other kernels for the in-house scans. We still observe a significant performance improvement measured by gray matter overlap with activations. However, this dataset is less robust compared with the HCP dataset.

### 4.2 Validation for self-supervised learning

Beyond subject-wise average accuracy, in this section, we further validate the self-supervised learning in three different directions. In Section 4.2.1, we numerically show that this method preserves the spatial correlation between voxels, unlike randomly shuffling that totally destroys it. In Section 4.2.2 we show an example from HCP dataset, and the detail process for the parameter optimization. In Section 4.2.3 we show the accuracy in subject-level analysis without normalization. In Section 4.2.4 we present a one-step argumentation technique, with application to 1D simulation. The one-step method has a major drawback that requires a large statistical average, which is difficult for high dimensional data like fMRI. Section 4.2.5 compares the performance using single shuffling (shuffling method 1) versus combined shuffling (shuffling method 1 and shuffling method 2). Section 4.2.6 further evaluates the convergence stability of the proposed surrogate-based optimization.

#### 4.2.1 Preservation of spatial correlation

**Fig. 11.**
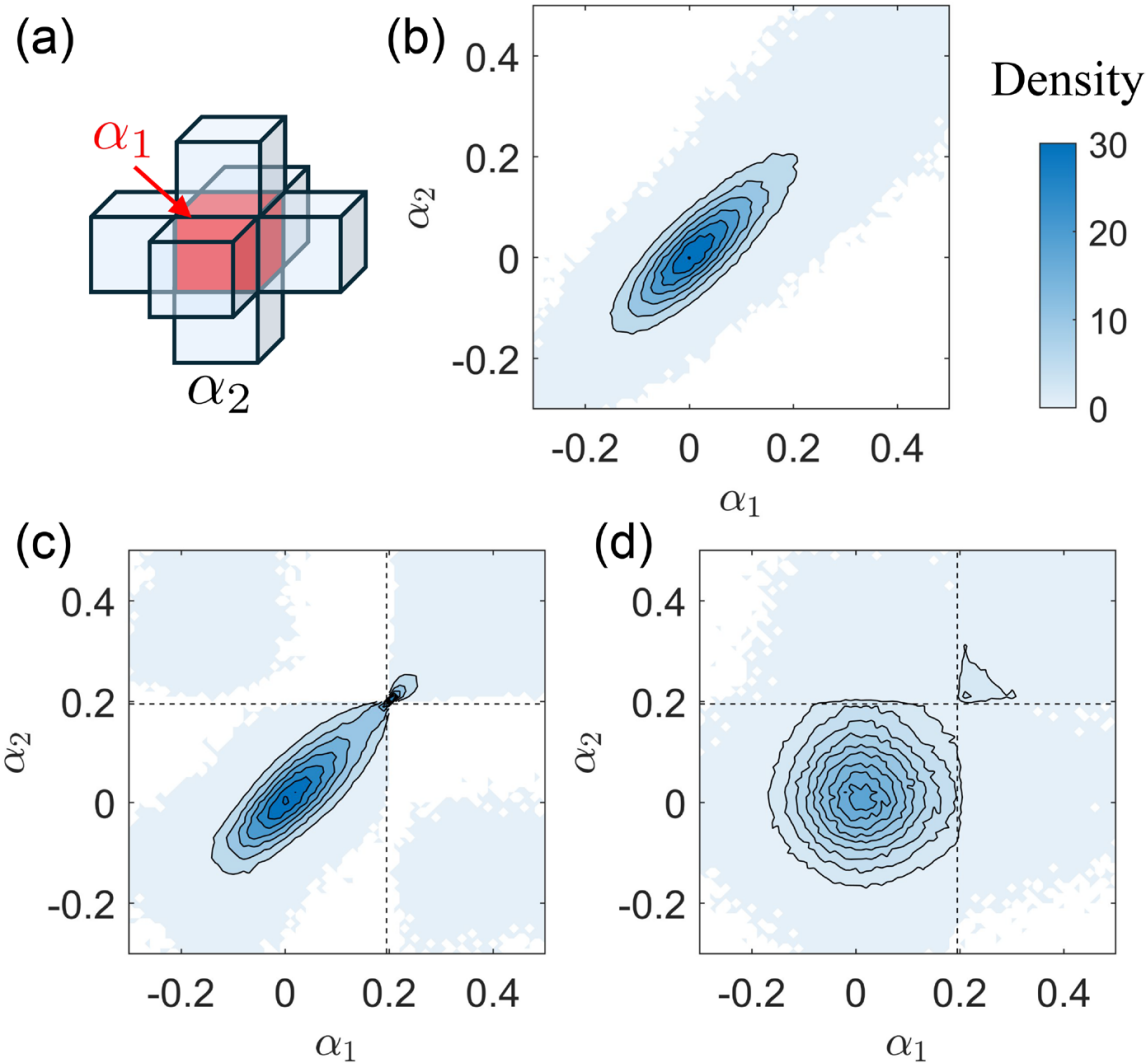
Nearest neighbor distributions for α before and after voxel shuffling. The results are taken from the HCP dataset using the linear kernel. There are no significant differences between distributions across different subjects, kernels, or BC methods. (a) Illustration of the center voxel $\alpha_{1}$ and its six neighbors $\alpha_{2}$. (b) Original nearest neighbor distribution. A large number of voxels located on the diagonal indicate that most neighboring voxels tend to have similar α values. (c) Nearest neighbor distribution after voxel shuffling, with the dashed line indicating the decision boundary. The majority of voxels remain on the diagonal, showing that the spatial correlation structure is preserved. (d) Random shuffling with the same cluster size for comparison, where the spatial correlation structure is destroyed.

We plot the joint probability of α between certain voxels and their neighbors. Consider a voxel labeled with $\alpha_{1}$ in Figure 11a, and its neighbor, labeled with $\alpha_{2}$. In Figure 11b, we show the probability density for different value pairs. The distribution is computed using one selected example from the HCP dataset using the linear kernel. We randomly generated $10^{6}$ pairs inside the brain to compute this map. The contour map is concentrated on a diagonal as the majority of voxels with similar α values tend to be neighborhoods. In other words, activated voxels tend to be a neighborhood with activated voxels, and similarly for non-activated voxels (Zhuang et al., 2017).

To characterize the distribution difference, we introduce the Kullback–Leibler (KL) divergence. Let $P_{\text{ori}}$ denote the original distribution shown in Figure 11b, and let $P_{\text{shu}ffl\text{e}}$ denote the distribution after shuffling. The KL divergence is then defined as

$$D_{\text{KL}}=\sum P_{\text{shu}ffl\text{e}}\text{ log}\frac{P_{\text{shu}ffl\text{e}}}{P_{\text{ori}}}.$$ (14)

Practically, a small number $10^{-6}$ is added in the denominator to avoid numerical overflow.

In Figure 11c, we plot the same distribution based on its new locations after shuffling. Compared with Figure 11a, the joint distribution does not change significantly, with $D_{\text{KL}}=0.6266$ , which proves that this shuffling does not destroy the spatial correlation between voxels. Finally, in Figure 11d, we plot the same distribution if we randomly shuffle the voxels in each cluster. The distribution looks totally different with $D_{\text{KL}}=1.6159$ . Moreover, our numerical results suggest that randomly shuffling the objective function is not deterministic, so the computational efficiency is not as good as with the current shuffling method.

#### 4.2.2 Consistency during optimization

The basic assumption is that, as optimization proceeds, activations with higher robustness exhibit better accuracy. This section aims to prove this consistency during optimization.

**Fig. 12.**
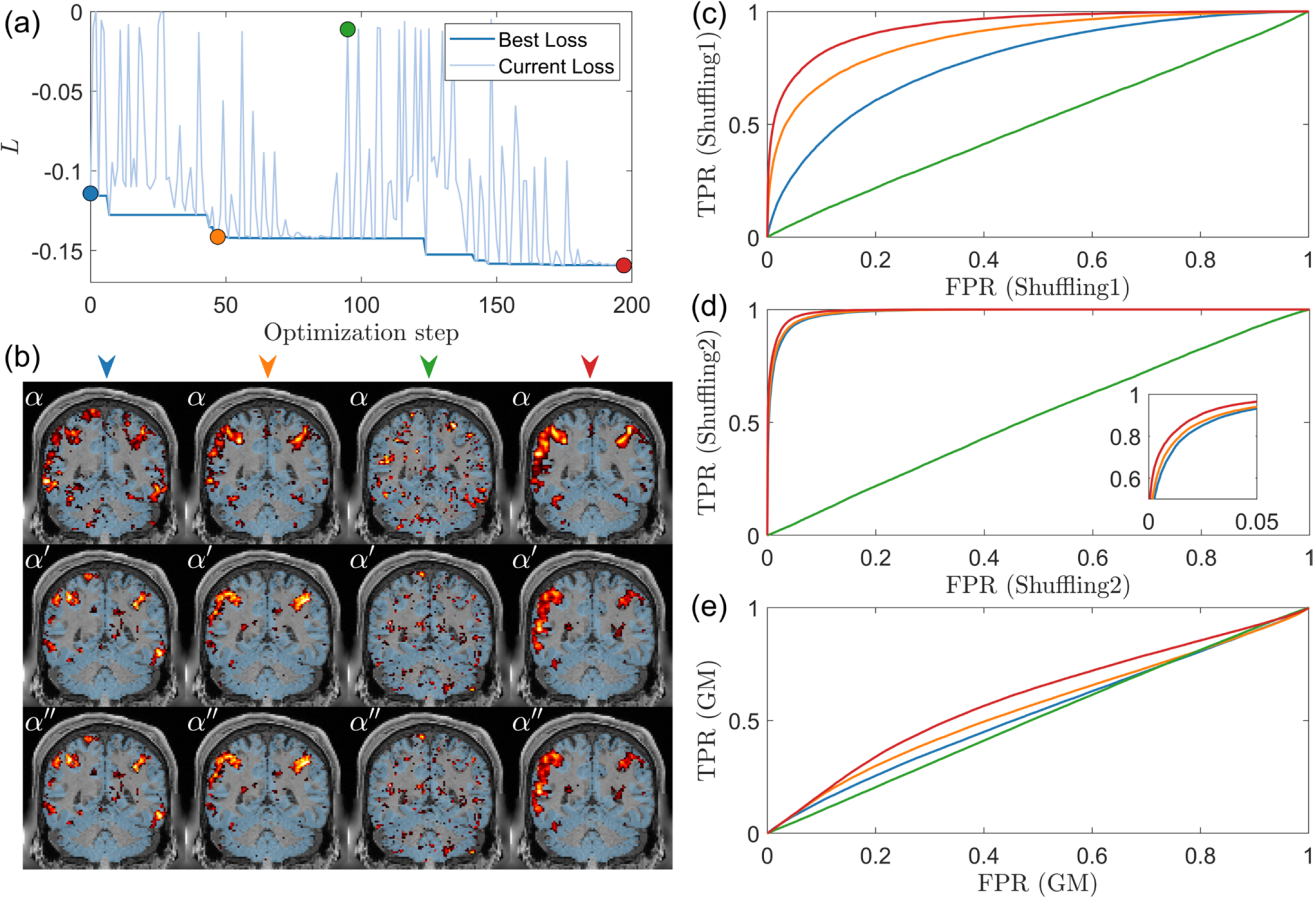
Example of parameter optimization for a selected subject from the HCP dataset using the mixed hyperbolic tangent kernel. For the same subject, Figure 6 presents the performance of other kernels. (a) Relationship between optimization steps and loss defined in Eq. (8). Since the optimization is not gradient based, significant fluctuations are observed. However, the loss stabilizes after approximately 170 steps. Four different points are highlighted by blue, orange, green, and red circles, corresponding to the linear kernel, an intermediate stage with improved performance, another intermediate stage with poor performance, and the mixed hyperbolic tangent kernel achieving the minimum loss, respectively. (b) Activation pattern visualization, with bright colors indicating the top 10% of voxels with high α values, overlaid on the gray-matter mask (blue) and the T1-weighted image. α represents the original activation pattern, while $\alpha^{\prime }$ and α″ indicate activation patterns after the first and second shuffling methods, respectively. The second shuffling method tends to produce more similar results since only voxels near the decision boundary are modified. (c) AUC curve comparison between α and $\alpha^{\prime }$ to evaluate the effect of the first shuffling method. (d) AUC curve comparison between α and α″ to evaluate the effect of the second shuffling method. Inset: a focused view in the top-left corner. (e) AUC curve for activation in gray matter. The results clearly show that activation patterns that are more robust to shuffling also exhibit greater overlap with gray matter, confirming the assumption for self-supervised learning during the optimization process.

Figure 12 shows the parameter optimization for one of the selected subjects from the HCP dataset for the mixed hyperbolic tangent kernel. A brief overview of our data processing pipeline is as follows:

1. Perform fMRI preprocessing without applying smoothing.
2. Select the smoothing method and kernel mapping function. We use the steerable filters in Appendix A.1, generated with FWHM = 4 mm.
3. Initialize certain parameters and run KCCA to obtain α. The initial parameters are chosen based on the results from the linear kernel: $b_{1}=10^{-3},\;b_{2}=0,\;c=0$ , and $\gamma =\gamma_{\text{Linear}}$ , where $\gamma_{\text{Linear}}$ is the optimal regularization parameter found for the linear method. A small $b_{1}$ ensures that optimization starts from the linear kernel.
4. Apply voxel shuffling twice based on α to generate ${\text{Y}}^{\prime }$ and ${\text{Y}}^{\prime }$. The voxel shuffling is performed before smoothing, with the second method modifying a relatively small number of voxels.
5. For each ${\text{Y}}^{\prime }$ and ${\text{Y}}^{\prime }$, map the data to the kernel space using the same kernel and parameters. Apply the KCCA algorithm again to establish the relationship in the kernel space, then use BC 1 to obtain activation maps $\alpha^{\prime }$ and α″ .
6. Evaluate Eq. (8) by comparing the augmented activation patterns with the original activation pattern.
7. Run an optimization algorithm to find the best parameters that minimize Eq. (8).
8. Once optimal parameters are found, evaluate the activation maps using the method described in Section 3.8.2.
9. Repeat the same computations for the GLM+GS model to generate the baseline results.

In Figure 12a, we present the loss defined in Eq. (8) as a function of the optimization step. The loss curve is not guaranteed to decrease smoothly because the optimization is performed using MATLAB’s surrogate optimizer rather than a gradient-based local solver. The surrogate method is a global derivative-free algorithm that alternates between constructing a surrogate model and evaluating candidate points within the bounded parameter space. As a result, the reported objective values can fluctuate during the search, even when the algorithm is progressing toward a better solution. By monitoring the best loss, the algorithm generally converges well after 170 steps. During optimization, we select four points, indicated by blue, orange, green, and red circles, representing the initial result from the linear kernel, an intermediate stage with improved performance, an intermediate stage with poor performance, and the best result found by the optimization algorithm.

Figure 12b displays the original activation pattern α alongside the augmented activation patterns obtained from two different shuffling processes. The arrows on the top indicate the locations where the activation pattern is extracted. To ensure consistency and comparability, we plot the voxels with the top 10% of α values. Comparing the results before and after shuffling, we observe that the second shuffling method preserves more activation structure since only voxels near the decision boundary are modified. Comparing different optimization stages, we observe an increase in robustness from the linear kernel to the mixed hyperbolic tangent kernel. However, at the green-labeled location, the spreading cluster-like activation does not correspond to meaningful results, leading to low similarity between the original and augmented activation patterns.

Figure 12c and d shows the ROC curves computed using activation patterns before and after shuffling. Specifically, Figure 12c compares α and $\alpha^{\prime }$ , generated using the first shuffling method, while Figure 12d compares α and α″ , generated using the second shuffling method. This type of curve has been referred to as the “apparent ROC” in previous studies (Zhuang et al., 2017). Similar to the observations in Figure 12b, the second shuffling method results in a relatively high AUC, with the lowest AUC observed at the green-labeled location and the highest AUC at the red-labeled location.

Figure 12e presents the gray matter overlap of activated voxels at each optimization stage. The results align with the shuffling robustness observations, confirming that our method successfully identifies the optimal parameter combination without requiring additional datasets.

#### 4.2.3 Subject-level validation

For the relationship between shuffling robustness and accuracy, Figures 4, 6, 8, and 10 demonstrate that this assumption holds at the subject-wise average. Here, we examine whether it also holds at the subject level. To clarify the results, we do not apply normalization and include GLM+GS for comparison.

**Fig. 13.**
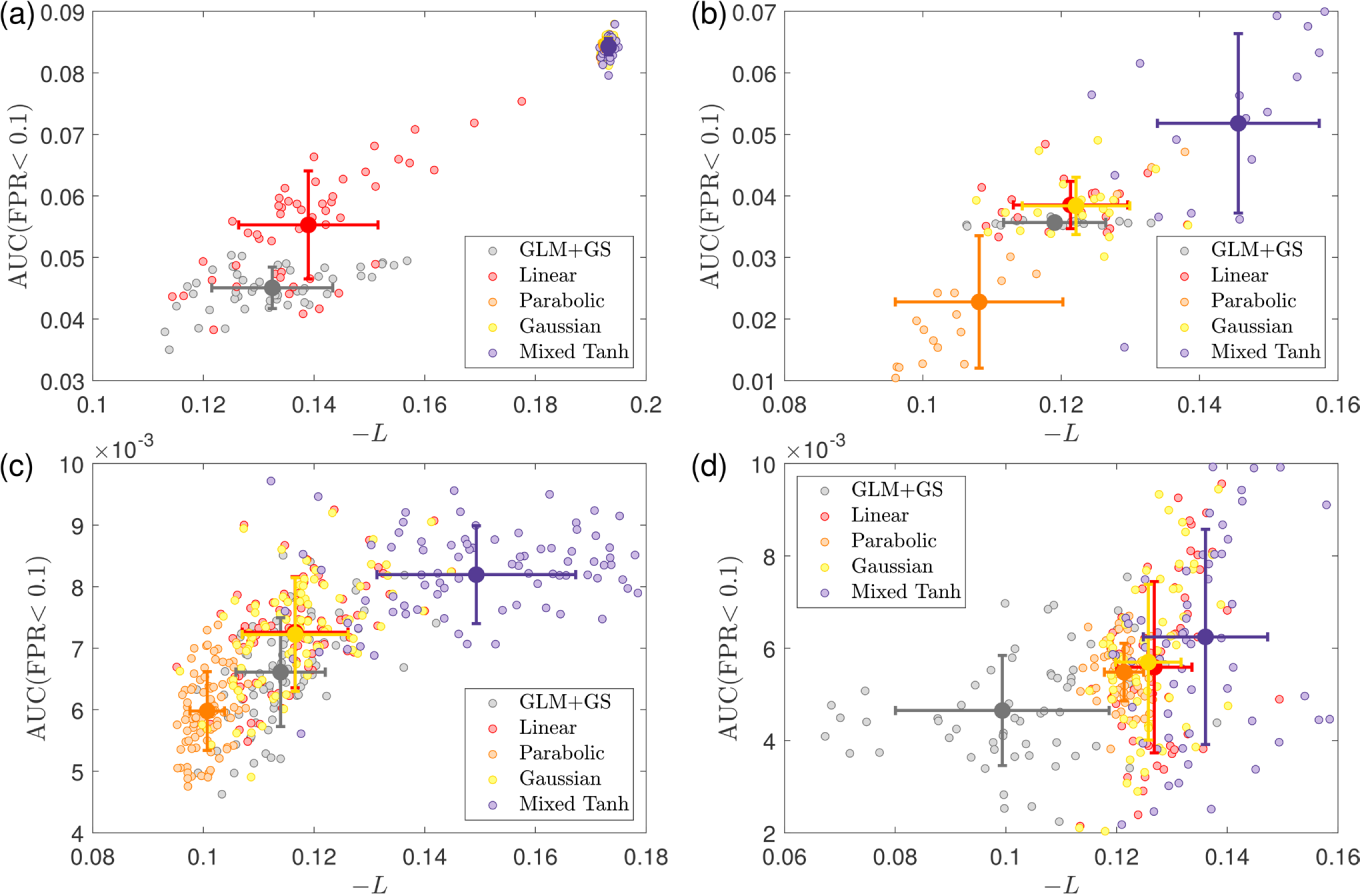
Subject-level results for accuracy versus shuffling robustness, where both metrics are unnormalized. The horizontal axis represents −ℒ , which is the summation of two different shufflings as defined in Eq. (8). The vertical axis represents unnormalized accuracy. The values correspond to the ground truth for (a) the first simulated fMRI dataset described in Section 4.1.1 and (b) the second simulated fMRI dataset in Section 4.1.2. For (c) the HCP dataset and (d) the in-house scans, the values correspond to activation overlapping with gray matter. Error bars are included for each method.

Figure 13 presents subject-level results for all four datasets analyzed in this study. Four kernels are selected: the linear kernel, the commonly used parabolic and Gaussian kernels, and the mixed hyperbolic tangent kernel proposed in this study. Figure 13a and b shows results for the two simulated datasets, measuring both shuffling robustness and accuracy, while Figure 13c and d displays results for the two task-fMRI datasets, evaluating shuffling robustness and activation overlays on gray matter. All methods are presented together with their mean and variance.

Figure 13a shows that in the first simulation, except for the linear kernel, all other kernels successfully capture the relationship with high accuracy and shuffling robustness. This observation aligns with Table 5, which reports normalized accuracy. In Figure 13b and c, a clearer separation between different kernels is observed, with an approximately monotonic relationship. Figure 13d presents results for the in-house scans, where GLM+GS does not perform well. Although the relationship is less pronounced, the trend remains consistent at the subject-wise average.

From a performance point of view, when comparing the two simulated datasets, it can be seen that the first simulated dataset with a nonlinear kernel performs much better than with a linear kernel. As shown in Figure 13a, the shuffling robustness −ℒ defined in Eq. (8) exceeds 0.18 , which is very close to the maximum value of 0.2 , indicating that the nonlinear kernel can effectively capture the relationship in this case. Overall, this simulation is relatively simple, and the task remains easy to learn. Fixed mappings such as linear and square kernels lack tunable parameters and, therefore, adapt poorly, whereas nonlinear kernels better capture the underlying structure.

For the two task-fMRI datasets, minimal preprocessing was applied and explicit motion regression was not performed to avoid introducing high-frequency artifacts. To further evaluate its effect, we regressed out the six motion parameters provided in the HCP dataset and analyzed the resulting power spectra. As described in Appendix A.6, motion regression reduces power below 0.05 Hz but increases power at higher frequencies. Therefore, a trade-off exists: while motion regression can suppress motion-related components, it may also alter the spectral distribution by increasing high-frequency artifacts.

#### 4.2.4 Comparison between one-step and two-step data augmentation

In Tables 5 and 6, we show that, in general, bounded kernels outperform unbounded kernels. Following previous work on noise contamination (Alam, 2014), we introduce an alternative augmentation approach based on noise robustness that does not require smoothing, to further provide evidence for this observation.

**Table 6.** Comparison between different back-reconstruction methods.

	fMRI simulation 1		fMRI simulation 2	
Kernel name	BC 1 (This study)	BC 2 (Han et al., 2025)	BC 1 (This study)	BC 2 (Han et al., 2025)
Linear	5.25%	5.25%	1.09%	1.09%
Parabolic	15.17%	2.92%	−18.57%	2.51%
Gaussian	15.20%	3.42%	0.49%	$\underline{1.65\text{\%}}$
Inverse	$\underline{15.19\text{\%}}$	3.41%	0.15%	1.35%
Bounded linear	15.06%	3.19%	3.11%	1.18%
Square	−6.88%	−1.34%	−5.99%	−0.03%
Tanh	14.89%	$\underline{9.79\%}$	$\underline{3.32\text{\%}}$	−14.82%
Mixed tanh	15.14%	11.90%	4.78%	1.10%
	HCP dataset		In-house scans	
Kernel name	BC 1 (This study)	BC 2 (Han et al., 2025)	BC 1 (This study)	BC 2 (Han et al., 2025)
Linear	0.55%	0.55%	4.47%	$\underline{4.47\%}$
Parabolic	−4.21%	0.57%	9.39%	3.24%
Gaussian	0.46%	1.06%	5.51%	3.95%
Inverse	0.35%	0.66%	$\underline{5.72\text{\%}}$	4.01%
Bounded linear	2.99%	0.70%	3.60%	6.38%
Square	3.62%	$\underline{3.86\text{\%}}$	3.53%	1.89%
Tanh	$\underline{4.54\text{\%}}$	1.21%	4.78%	2.41%
Mixed tanh	6.50%	5.34%	5.67%	2.93%

The performance improvement ratios are defined using Eq. (9) or Eq. (10) for the total ROC. For each dataset and each back-reconstruction method, the best-performing method is shown in **bold**, and the second-best method is shown as underlined.

Using 1D simulation shown in Section 2.7, once all data are generated, the baseline model is chosen to be the GLM. Specifically, it computes the correlation at each location, represented by $\alpha \in {ℜ}^{Q\times 1}$ , and then compares it with the ground truth to generate the ROC curve. Similar to fMRI data, we conduct kernel analysis without smoothing. To simplify our results, we consider only the linear, parabolic, Gaussian, and mixed hyperbolic tangent kernels. For kernel methods without smoothing, the transformation can be interpreted as treating A as an identity matrix in Eq. (5).

**Fig. 14.**
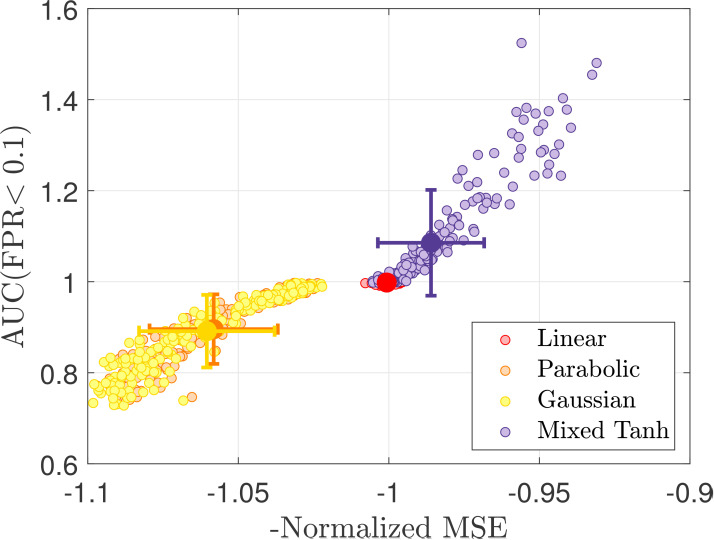
Results for the 1D simulated data. The robustness to noise contamination and the accuracy of four selected kernels are evaluated without applying any smoothing. Shuffling robustness (x-axis) is quantified using Eq. (7), while accuracy is measured by the AUC under the constraint FPR <0.1 . Both metrics are normalized relative to the GLM to obtain relative ratios. Since a lower MSE indicates higher robustness, a negative sign is applied to the x-axis. Overall, increased robustness is generally associated with higher accuracy. Among the kernels considered, the mixed hyperbolic tangent kernel exhibits both the highest robustness and the highest accuracy.

Figure 14 presents subject-level performance for four selected kernels: linear, parabolic, Gaussian, and the mixed hyperbolic tangent. Each dot represents the result for an individual subject, with the mean and variance for each method also displayed. The (1, 1) point indicates performance equivalent to that of GLM.

Figure 14 shows that for the linear kernel, nearly all results fall close to the (1,1) point, indicating that in the absence of smoothing, the linear kernel behaves similarly to GLM. In contrast, the mixed hyperbolic tangent kernel has most of its points in the upper-right corner, suggesting that it improves both accuracy and robustness to noise contamination. However, unbounded kernels such as the parabolic kernel perform poorly, with most points located in the bottom-left corner.

These empirical results demonstrate that the mixed hyperbolic tangent kernel is more resilient to noise contamination than the linear kernel, consistent with previous analyses on bounded kernels (Alam, 2014). This finding provides an alternative perspective on the kernel selection problem. However, applying this method to task-fMRI data is challenging, as it requires extensive statistical averaging during the optimization process, making it impractical for large-scale fMRI datasets.

#### 4.2.5 Comparison of single and combined shuffling methods

We additionally report results obtained using only the first shuffling method. Due to computational constraints, this comparison is conducted on a subset of 40 subjects from the HCP dataset with smaller subject IDs, corresponding to approximately 50% of the full dataset, under the first back-reconstruction method.

We compare the performance of using a single shuffling method with that of using two shuffling methods. For the linear kernel, the first shuffling method achieves an accuracy of 9.86%, while two shuffling methods yield 9.84%, indicating nearly identical performance. For the mixed hyperbolic tangent kernel, using one shuffling method results in an accuracy of 24.64%, whereas using two shuffling methods improves the accuracy to 25.21%.

The primary motivation for adopting two shuffling methods is robustness. When only the first shuffling method is used, we observe two subjects whose performance drops below the GLM+GS baseline; in contrast, when both shuffling methods are employed, all subjects perform above the baseline. This suggests that incorporating multiple shuffling methods can benefit more complex kernels, such as the mixed hyperbolic tangent kernel, and improve cross-task generalization.

#### 4.2.6 Optimization stability analysis

We further evaluate the stability of the surrogate-based optimization procedure. This analysis is conducted on a subset of 40 subjects from the HCP dataset with smaller subject IDs, using the first back-reconstruction method, to remain consistent with the experimental setup in Section 4.2.5.

To assess convergence behavior, we extend the number of optimization steps by 50% beyond the values reported in Table 3, and compute the relative change in the objective function ℒ. For the linear kernel (15 to 22 steps), the relative change in ℒ is approximately 0.3% . For the mixed hyperbolic tangent kernel (200 to 300 steps), the relative change is approximately 0.7% .

These changes are substantially smaller than the performance differences observed across kernels (greater than 20% ), indicating that the selected optimization budgets are effectively stable. This empirical evaluation supports the use of fixed optimization steps in our surrogate-based framework.

### 4.3 Comparison between BC methods

In this section, we compare the new BC method (denoted by BC 1) proposed in this study with the previous method, denoted as BC 2 (Han et al., 2025). For a fair comparison, both methods are applied to the same fMRI data after preprocessing. During the optimization process, each kernel in both methods utilizes the same parameter ranges specified in Table 3 and follows the same optimization steps. For the linear kernel, both BC methods produce identical results, with differences appearing only for nonlinear kernels.

Table 5 presents a comparison of accuracy with FPR <0.1 across the four datasets analyzed in this study. For each dataset column, the best-performing kernel is highlighted in bold, while the second-best method is underlined. Overall, the BC 1 outperforms BC 2 for all the datasets, the increased ratio is more obvious for simulated dataset than for the task-fMRI dataset.

In particular, for the first simulated dataset, the new method achieves better results compared with the previous one, with performance improvements ranging from approximately 86% to 89%. Although the mixed hyperbolic tangent kernel is not numerically the best in this case, its improvement is very close to that of the best-performing kernel. For the second simulation and the two task-fMRI datasets, performance differences across kernels are more pronounced, with the mixed hyperbolic tangent kernel together with BC 1 emerging as the best method.

We also report the total AUC obtained in this study, as summarized in Table 6. In general, three observations can be made. First, the improvement rate for the overall AUC is relatively small. Second, except for the in-house scans, kernels that perform well for FPR <0.1 also tend to show better performance for the overall AUC. Third, BC 1 still outperforms BC 2. This further demonstrates the superiority of BC 1, regardless of the activation threshold or other considerations during evaluation.

### 4.4 Activations within a region of interest

Activation in gray matter characterizes the overall performance, but it is not sufficiently specific for a given task. Here, we examine activation overlap with the ROI, whose definition is shown in Section 3.8.4. Because the mask is relatively small and induces a large variance, we report only the t-test results aggregated across all subjects.

In Table 7, we list four types of comparisons: kernel-based methods versus GLM+GS, as well as the mixed hyperbolic tangent kernel versus the linear kernel. We find that, except for the in-house scans comparing the linear kernel with GLM+GS, all other comparisons show statistical significance, indicating that the proposed method increases activation within the desired region.

**Table 7.** Statistical test for activation within the region of interest.

Method Comparison	HCP dataset	In-house scans
Linear vs GLM+GS	<0.001	0.1030
Tanh vs GLM+GS	<0.001	0.0358
Mixed tanh vs GLM+GS	<0.001	<0.001
Mixed tanh vs linear	<0.001	0.0089

One-sided paired t-test p-values comparing the $r_{\text{ROI}}$, defined in Eq. (12), among the GLM+GS, linear kernel, hyperbolic tangent kernel, and mixed hyperbolic tangent kernel.

### 4.5 Eigendecomposition

While the results from Section 4.1, Section 4.3, and Section 4.4 indicate the superior performance of the mixed hyperbolic tangent kernel, these metrics focus more on performance, not the kernel itself. We propose using the $r_{\text{Corr}}$ defined in Eq. (13). The advantage is that this quantity is purely determined by the kernel and is independent of back-reconstruction or parameter optimization, which makes the comparison clearer. To narrow down our comparison, we examine $r_{\text{Corr}}$ in two ways: kernels with the same complexity but different convergence properties and kernels with the same convergence properties but different complexity.

We compare results among kernel mappings with the same complexity (one unknown parameter). Several details are listed as follows. First, we note that the hyperbolic tangent kernel proposed in Table 3 has two parameters. To address this, we propose a reduced tanh with only one parameter by removing the constant term: $K_{Y}\left({\tilde{\text{Y}}}_{i},{\tilde{\text{Y}}}_{j}\right)=\text{tanh}\left(b{\tilde{\text{Y}}}_{i}{\tilde{\text{Y}}}_{j}^{T}\right)$. Second, the maximization in Eq. (13) is performed via grid search with 200 discretizations in each dimension using equal spacing. Third, for comparison, we also include the linear kernel. In conclusion, from a complexity perspective, both the parabolic kernel and the reduced tanh have one parameter, whereas the linear kernel has no tunable parameter. The reduced tanh is bounded, the linear kernel is unbounded but first order, and the parabolic kernel exhibits quadratic divergence.

Table 8 shows the statistical significance of the reduced hyperbolic tangent kernel over unbounded kernels such as the linear and parabolic kernels. We also observe that, in three of the five tasks, the linear kernel outperforms the parabolic kernel. Moreover, across all five tasks, the reduced hyperbolic tangent kernel shows better performance than both the linear and parabolic kernels. This indicates that the bounded kernel encodes richer information compared with unbounded kernels.

**Table 8.** Statistical tests for eigendecomposition under fixed mapping complexity.

	Linear vs Parabolic	Reduced Tanh vs Linear	Reduced Tanh vs Parabolic
1D simulation	<0.001	<0.001	<0.001
fMRI simulation 1	<0.001	<0.001	<0.001
fMRI simulation 2	0.239	0.002	0.005
HCP dataset	<0.001	<0.001	0.002
In-house scans	0.532	<0.001	<0.001

One-sided paired t-test p-values comparing the kernel decomposition measure $r_{\text{Corr}}$, defined in Eq. (13), ` hyperbolic tangent kernels.

Next, we examine the effect of unknown parameters under the same boundedness property. This type of analysis is motivated by previous studies of mixed kernels (Zhu et al., 2012). We compute $r_{\text{Corr}}$ among reduced hyperbolic tangent, hyperbolic tangent, and mixed hyperbolic tangent. Since increasing the parameters under this setup always increases the maximum value in Eq. (13), the corresponding t-test does not provide additional insight. Therefore, we instead compare the raw values to assess this effect.

Table 9 reports the $r_{\text{Corr}}$ values for different kernels. In the 1D simulation, $r_{\text{Corr}}$ is substantially higher, likely reflecting the relatively simple structure of this problem compared with fMRI simulations or task-fMRI data; in more complex settings, kernels with additional terms can encode richer information and thus yield higher $r_{\text{Corr}}$ . However, whether increasing kernel complexity—and consequently $r_{\text{Corr}}$ —consistently translates into improved activation accuracy remains unclear. This issue is further discussed in Section 5.1.4 and Section 5.3.1.

**Table 9.** List of the average *r*_Corr_ among different kernels.

	Reduced Tanh	Tanh	Mixed Tanh
1D simulation	0.8921	0.8933	0.8936
fMRI simulation 1	0.4008	0.4715	0.4886
fMRI simulation 2	0.3197	0.3836	0.4212
HCP dataset	0.2932	0.3492	0.3622
In-house scans	0.4443	0.4775	0.4947

List of the average $r_{\text{Corr}}$, defined in Eq. (13), among the reduced hyperbolic tangent, hyperbolic tangent, and mixed hyperbolic tangent kernel.

## 5 Discussion

### 5.1 Self-supervised learning

#### 5.1.1 Comparison with computer vision

Compared with the contrastive learning framework proposed in T. Chen et al. (2020), a commonality is that both methods use data augmentation techniques to generate supervisory examples. However, there are also several key differences.

First, in CNN-based classification problems, class labels are treated as the ground truth. Self-supervised learning primarily focuses on pre-training, followed by a fine-tuning stage to determine the model parameters. In contrast, our work utilizes self-supervised learning to determine unknown parameters, as the KCCA algorithm maximizes correlation in the kernel space, which is equivalent to the supervised learning approach used in previous work.

Second, in computer vision, a one-step approach—data augmentation without using model outputs—is usually adopted (Mo et al., 2021). In our study, we primarily focus on a two-step voxel shuffling. The trade-off is that one-step augmentation is much more efficient for GPU parallelization, whereas the two-step approach avoids randomness and requires fewer statistical averages. Due to practical computational considerations, we only use the two-step method for fMRI.

Third, in computer vision, both positive and negative pairs are used (T. Chen et al., 2020). In our study, however, only positive pairs are considered, where we maximize the similarity between the original data and the augmented data. While it is possible to generate negative pairs in fMRI data, such as by shuffling the time domain ${\text{v}}_{\text{Y}}$ to determine the activation threshold, we found this process requires extensive statistical averaging. As a result, we did not implement negative pairs.

Another related topic is adversarial training (Goodfellow et al., 2014), where adversarial examples are intentionally introduced during training to enhance robustness. Some adversarial examples are generated using similar augmentation techniques compared with contrastive learning (Cohen et al., 2019; Liu et al., 2019; Prakash et al., 2018). However, the current adversarial training still requires labeling examples. In other words, the loss function is evaluated using ground truth and the output from adversarial examples. In our study, we observe that maximizing the similarity between the original and augmented output is already enough for the model to generate accurate activation. Therefore, we call the method self-supervised instead of adversarial training.

#### 5.1.2 Comparison with previous optimization techniques in an fMRI study

Compared with the voxel shuffling method used previously (Zhuang et al., 2017), our method can shuffle the entire fMRI dataset at once, making it suitable for global methods such as KCCA.

Compared with previous work that used resampled resting-state fMRI data as a reference (R. R. Nandy & Cordes, 2003, 2004; Yang et al., 2018), we found that maximizing the difference between task-fMRI and resampled resting-state fMRI data does not yield stable results for nonlinear cases. One possible reason is that nonlinear kernels introduce too many opportunities to create differences between task and resampled resting-state fMRI data, making purely numerical comparisons unstable. Our self-supervised learning approach also reduces the data used during the optimization extraction process.

An alternative approach for task-fMRI is to define the objective function based on activation overlapping with gray matter, a method similar to that used in deep CCA studies (Yang et al., 2025). However, we find that for global methods such as KCCA, maximizing activation within gray matter may yield better numerical values but does not consistently align with the expected results observed with the linear kernel. Instead, optimizing based on voxel shuffling provides a more reliable solution. Moreover, optimization via voxel shuffling is more convenient, as it does not depend on gray matter labels. The success of simulated data also indicates its broad applicability to the nonlinear KCCA problem.

#### 5.1.3 Assumption validation

The first assumption is that we aim to create positive pairs. Therefore, we want to maintain the spatial relationship after shuffling. As shown in Figure 11, the nature of fMRI analysis is that voxels with similar activation tend to be spatially close to each other. As a result, when we shuffle the voxel locations, the order is reversed, the assumption remains valid, and the spatial correlation is maintained after shuffling.

The second assumption in self-supervised learning is that activation patterns with high robustness to shuffling also exhibit high accuracy. In other words, the assumption is about the cross-task generalization problem. From Figures 4, 6, 8, and 10, subject-wise average shows a monotonic relationship between the shuffling robustness and gray matter overlap. A similar relationship also appears in Figures 12 and 13.

We also note that the performance of self-supervised learning is closely related to the back-reconstruction method. For example, when we compute the same relationship using BC 2, the association is weaker than that observed with BC 1.

#### 5.1.4 Strengths, limitations, and further work

The advantages of the proposed self-supervised learning method are as follows. First, from a computational perspective, it does not require an additional dataset, such as resting-state fMRI data or simulated data. Second, the method prevents overfitting across multiple simulated and task-fMRI datasets and demonstrates robustness at the subject level. Third, we show that the method is valid across different directions, offering insights into its internal mechanisms based on voxel shuffling, beyond simple accuracy improvements, which is an important aspect of self-supervised learning in computer vision (Tamkin et al., 2023).

We also note that several aspects remain unresolved. In particular, what constitutes the optimal shuffling method is still an open question. Identifying such a method is challenging due to the substantial computational cost involved. Based on our empirical observations, we summarize several heuristic findings below. First, combining statistical averages across different shuffling methods can improve performance. Second, from a general complexity analysis, more complicated kernels benefit more from statistical averaging during shuffling. Third, we only tested combinations of different shuffling methods with equal weights, following a common first-step strategy in many self-supervised learning frameworks (e.g., Swin Transformer (Tang et al., 2022)). Although shuffling along boundaries results in smaller voxel displacements than shuffling all voxels and typically yields a slightly higher AUC, the variation in AUC across kernel mappings is small, as the optimization primarily depends on relative changes rather than absolute values. Two shuffling methods are combined with equal weights.

Second, it remains unclear whether one-step shuffling exists for the activation detection problem. For example, in Section 4.2.4, we find that augmentation without using model output exists, but it is generally hard to implement for task-fMRI due to computational limitations. One possible direction is to apply augmentation at the level of image patches (Tang et al., 2022).

Third, it is worth considering whether the current self-supervised learning approach can be extended to other problems. For example, in a typical classification-based problem, one could compute a heat map defined by voxel importance to generate supervisory examples, encouraging the model to produce consistent predictions (Rasmussen et al., 2011; Simonyan et al., 2013). This approach could also be combined with resampling techniques (Laird et al., 2004), which are typically used to break spatial correlations and generate negative pairs.

### 5.2 Back-reconstruction methods

#### 5.2.1 Comparison with deep learning-based methods

Compared with deep learning methods, our approach relies on a more mathematically grounded back-reconstruction framework. Deep learning-based models, such as the Swin Transformer, also employ self-supervised learning for training (P. Kim et al., 2023; Kwon et al., 2025; Tang et al., 2022) and have the capacity to generalize across multiple tasks as foundation models (Karimi, 2024). Here, we summarize several advantages and disadvantages in comparison with deep learning.

First, in terms of design complexity, the primary source of complexity in our nonlinear KCCA framework lies in the mathematical design of the kernel map and the back-reconstruction method. Each kernel and back-reconstruction scheme has a well-defined but distinct mathematical form, and the overall performance depends strongly on these modeling choices. By contrast, in deep learning, the functional form of the model is typically fixed, while variability mainly arises from the network architecture and training configuration (e.g., network depth, width, normalization layers, learning schedules, and regularization strategies), which inevitably increases the tuning burden and may complicate interpretation.

Second, in terms of computational speed, the training phase for deep-learning-based methods typically requires expensive computational resources, such as GPUs, especially for high-dimensional fMRI data. Deep learning is also usually based on very large datasets. An advantage is that deep learning often provides faster inference once the model is trained, as no parameters need to be re-estimated. For nonlinear KCCA, a disadvantage is that parameters need to be evaluated for each subject. However, its advantages are that it requires fewer computational resources, as computation can be performed at the subject level and only CPUs are needed.

Third, while deep learning models are often equipped with post hoc interpretability tools such as saliency maps or heat maps (Selvaraju et al., 2017; Smilkov et al., 2017; B. Zhou et al., 2016), which are conceptually similar to the second back-reconstruction strategy in nonlinear KCCA, their interpretability remains limited because the relationship between the learned representation and the design signal is implicitly encoded within a complicated network architecture. In contrast, KCCA explicitly models the relationship between two domains through well-defined mathematical structures, enabling direct interpretation of how information is shared across modalities in both linear and nonlinear settings.

Another way to view interpretability is that, while deep learning demonstrates strong performance for many problems, it often suffers from issues such as covariance shift across domains, overfitting when the number of subjects is limited, or dimensionality shift. In contrast, our nonlinear KCCA maintains a clearly defined mathematical structure, making it more flexible for different problems without requiring substantial changes to the model.

#### 5.2.2 Comparison with a previous back-reconstruction method

We give further analysis of the new proposed back-reconstruction method and the previous method (Han et al., 2025). First, for the mathematical properties, both methods can reduce to linear kernel results, as proven by Appendix A.5. Both formulas can be derived from Eq. (4), as shown in Appendices A.3 and A.4. BC 2 has more assumptions, which are less rigorous compared with the method proposed in this study.

Second, both methods preserve the sign of the activation. This means that α obtained from the BC method can be treated as the effective correlation by the kernel method, making it generally suitable for a wide range of tasks with activated or de-activated regions. This equivalence is further supported by the similarity of activation patterns.

Third, for the computational speed, both BC methods can be converted to matrix operations with given ${\text{v}}_{\text{Y}}$. The time complexity for both methods is O(QT), but BC 1 is more efficient due to its simpler formula, especially for complicated kernels. Practically, other operations such as reshaping could minimize their differences. We observe approximately twice the efficiency for BC 1 in computation time in the back-reconstruction.

From a performance point of view, this method offers several advantages. First, subject-wise average results indicate that this method improves accuracy compared with voxel importance, as demonstrated across multiple simulated and task-fMRI datasets. Second, it exhibits lower variance than previous methods. Using the mixed hyperbolic tangent kernel on task-fMRI data as an example, in the HCP dataset, we observed only one subject with performance lower than the baseline, whereas this number was five with the previous method. For the in-house scan, the current method resulted in 12 cases below the baseline, compared with 20 with the previous method.

There are also general limitations of the new back-reconstruction method together with the global method. First, similar to Han et al. (2025), local information is missing, making the p value typically used for fMRI activation analysis difficult to define. Second, global methods such as KCCA extract relationships across the whole brain, and it is unclear how spatial diversity may affect the results, such as regional differences in hemodynamic response function (HRF) characteristics (Erhardt et al., 2012; Glover, 1999; Lindquist et al., 2009). Developing an optimal approach to address this spatial heterogeneity problem is still an open question.

### 5.3 Kernel selection strategies

One of the key questions is which kernel is most efficient for extracting information from fMRI data. A rigorous answer is difficult because this process involves multiple steps, including kernel selection, parameter estimation, and back reconstruction. We attempt to address this question from three perspectives: performance on simulated and task-fMRI data, augmentation robustness, eigendecomposition viewpoint on which kernels capture information related to the target signal, and insights from prior work.

#### 5.3.1 Bounded vs unbounded kernel

The first property we observe is the distinction between bounded and unbounded kernels. Among the eight kernels analyzed in this study, the parabolic kernel performs poorly, showing the lowest accuracy in half of the tasks. The linear, Gaussian, and inverse kernels perform similarly. The bounded linear and square kernels exhibit inconsistent results. Finally, the best-performing group consists of the hyperbolic tangent and mixed hyperbolic tangent kernels. Moreover, robustness to data augmentation further supports this finding, as the activation patterns from the mixed hyperbolic tangent kernel exhibit the highest robustness across multiple datasets, regardless of whether one-step or two-step augmentation is used.

In the eigendecomposition, we found that the parabolic kernel is usually hard to encode information from the data. Specifically, under the same model complexity with one parameter, across all datasets, the parabolic kernel is composed of eigenvectors that have small correlations with the target signal. In contrast, the hyperbolic tangent kernel contains more information, exceeding the linear and parabolic kernels with statistical significance.

One possible explanation is that bounded kernels reduce the effect of noise self-correlation. In KCCA applied to fMRI, kernel mapping embeds the spatial dimension, where large diagonal elements often arise due to noise self-correlation. A bounded kernel mitigates these large elements through continuous mapping, leading to a more balanced matrix structure that facilitates information extraction. Another perspective supporting this explanation is that bounded kernels are generally more robust to noise contamination. As demonstrated in Section 4.2.4, using an alternative data augmentation technique in 1D without smoothing, the results indicate that bounded kernels, such as the mixed hyperbolic tangent, yield consistent performance in the presence of noise contamination. This noise-robustness property is consistent with previous studies (Alam, 2014; S.-Y. Huang et al., 2009).

#### 5.3.2 Single vs mixed kernel

Another observation relates to the unique properties of the mixed hyperbolic tangent kernel. This kernel combines both linear and nonlinear terms and, in certain special cases, can reduce to a purely linear form. Under similar bounded mappings, having more terms generally leads to greater representational flexibility. The first simulated dataset clearly shows that, when flexibility is lacking, the kernel mapping may not be able to fit the data, which in turn leads to poor performance for mappings that cannot capture the underlying structure, such as the linear or square kernels.

From the eigendecomposition perspective, the mixed kernel generally leads to larger $r_{\text{Corr}}$ values than single kernels. Because this relationship is evident, we do not perform additional statistical tests.

Our observations partially align with previous research on mixed-kernel analysis (Zhu et al., 2012), with the difference that, in prior studies, “mixed” referred to combinations of bounded and unbounded kernels.

### 5.4 Computational cost

The computational costs for this study can be divided into two parts. One is from original fMRI to kernel space, which takes $O\left(T^{2}Q\right)$ time complexity. Back-reconstruction process, transferring the data from the kernel space to the original space, takes O(TQ) time complexity. Even though the new back-reconstruction method is much more efficient in the second step, it does not totally avoid the time complexity generated by the mapping. Another difficulty is that the total problem is relatively large, as it contains four datasets, two methods, and eight different kernels.

As a practical example, one subject from the HCP dataset has dimensions 7×390×91×109×91 . Here, the first dimension 7 represents the number of spatial filters, the second corresponds to the time length, and the last three dimensions denote the spatial coordinates in MNI space. Storing these data in float32 precision requires approximately 10 GB of memory when using seven steerable filters. A typical commercial GPU, such as the NVIDIA GeForce RTX 3090 with 24 GB of memory, therefore, usually requires more than one GPU card to enable parallel processing, which in turn requires compatible hardware to operate them.

Due to the high dimensionality of fMRI data, a two-step approach may be preferable. These methods usually incorporate information from previous outputs, thereby introducing less randomness. The advantage is that less parallelism is needed; for example, we show that with only one or two shuffling, decent accuracy can be achieved. The disadvantage is that multi-step methods require a relatively complex design. In practice, on our workstation with an Intel Xeon w9-3475X CPU and 128 GB of memory, two subjects can be processed concurrently, so the mixed hyperbolic tangent kernel using the first back-reconstruction method requires approximately 6 hours of effective computation per subject.

### 5.5 Hyperparameter used in this study and validation

In Table 10, we list all the hyperparameters used, and their validation is described below. Thresholds used for simulated data generation are displayed in Table 2. For Simulation 1, similar activation patterns are adopted as those in Yang et al. (2020). Based on our practical experience, there is considerable flexibility in the choice of simulated data.

**Table 10.** List of hyperparameters in nonlinear KCCA, with detailed explanation and validation shown in Section 5.5.

Name	Value
fMRI simulation	Listed on Table 2
Smoothing level	FWHM =4 mm
Kernel mapping	Listed on Table 3
Activation (voxel shuffling)	Top 10%
Reduce false positive	FPR <0.1
Shuffling method 1	$Q_{1}=Q_{\text{Non}}$ , $Q_{2}=Q_{+}$
Shuffling method 2	$Q_{1}=Q_{2}=0.5Q_{+}$
Gray matter	Probability >0.5
Activation (plotting)	p<0.05

The threshold appearing in Method 3.2 specifies FWHM =4 mm with seven steerable filters. Except for the 1D simulation in Section 2.7, all fMRI data, including both task-based and simulated data, use a 4 mm smoothing filter. The same smoothing level and steerable filters are reported in Yang et al. (2018), and a similar smoothing level is used in Cordes et al. (2012).

For nonlinear kernel parameters shown in Section 3.4, since both the linear and square kernels are normalized to have unit standard deviation, the mapping magnitude is chosen to match this scale. The selected range of γ covers a relatively large region. As stated in Section 3.7.2, optimization stability was assessed empirically by extending the optimization steps by 50%; the resulting relative change in ℒ was below 1%, much smaller than the typical difference between kernels (greater than 20%).

The threshold appearing in Method 3.8 is based on the assumption that activation occupies approximately 10% of the total voxels within the brain. A similar metric is used in Yang et al. (2020). Another related consideration is reducing false positives in activation analysis. While a commonly used threshold of FPR <0.1 is adopted, this criterion indicates that we expect the activation to remain relatively small (Skudlarski et al., 1999). As a result, we choose this value as the shuffling threshold. Two further validations are provided. First, for the first simulated dataset, the activation proportion is not exactly 10% , but typically ranges from 9% to 11% . Nevertheless, the performance improvement is evident. Second, even when shuffling is performed using 10% of the voxels, performance evaluated using entirely different metrics—AUC with FPR <0.1 or the total AUC—shows clear improvement across different datasets.

The two shuffling thresholds are based on the activation thresholds described above. As stated in Section 5.1.4, this choice is mainly empirical due to high computational cost.

The threshold for gray matter is set to 0.5. We find that when this threshold is perturbed by 10% , from 0.45 to 0.55 , the relative change in the number of voxels is less than 3% . Therefore, we believe that this threshold selection does not have a major influence on the results.

One related issue is the use of p<0.05 to determine activation for plotting. This value is chosen solely for visualization purposes and does not affect the accuracy.

### 5.6 Subject- and group-level activation pattern

This section compares our subject-level analysis with the previous group-level analysis. First, all parameter optimization in this study is performed at the subject level without data separation. Here, “overfitting” refers to the case where parameters learned from nonlinear mappings focus solely on producing artificially large correlation values, while failing to recover biologically meaningful activation patterns.

Second, because activation is computed only at the subject level, the averages reported in this study are subject-wise averages. This differs from previous group-level analyses, in which fMRI data from the same group are concatenated along the time domain and activation is then computed at the group level. Notably, although the nonlinear KCCA algorithm has the capacity to perform such group-level computations, concatenating all data would require a very large amount of memory.

Compared with traditional group-level analysis, hippocampal and medial temporal lobe (MTL) activation in MCI shows a non-monotonic pattern rather than a simple reduction relative to normal aging. Several studies report reduced MTL or hippocampal activation in MCI during encoding or retrieval, consistent with neurodegenerative dysfunction (Johnson et al., 2004; Machulda et al., 2003; Petrella et al., 2006). In contrast, other studies demonstrate paradoxical hyperactivation in early or less impaired MCI, particularly during associative encoding tasks, despite minimal volumetric loss (Celone et al., 2006; Dickerson et al., 2005; Hämäläinen et al., 2007).

By simply comparing hippocampal activation using the ROI defined in Section 3.8.4 and evaluating the t-test between normal and MCI groups, both groups are considered across two tasks and two contrasts, leading to an effective sample size of 32 cases per group. The hypothesis is that hippocampal activation in the normal group is larger than that in the MCI group. The p-value for the linear kernel is 0.120 , while it is 0.204 for the mixed hyperbolic tangent kernel, with neither reaching statistical significance. Therefore, our conclusion is that, in this study, we did not identify statistically significant differences between groups. Note that these results are based on subject-wise averages rather than a traditional group-level analysis.

For the HCP dataset, a similar analysis could be applied to examine gender-related differences in activation (Schmidt et al., 2009). However, as our study primarily focuses on methodological development, with activation extracted at the subject level, even if such differences were present, we would not expect them to affect our overall conclusions.

### 5.7 Sign effect in activation analysis

A critical question is whether to retain the sign of α during activation analysis. In previous GLM studies (Friman et al., 2003), the sign is preserved to indicate that only signals positively correlated with the effective design signal are selected. Conversely, earlier local CCA studies (Zhuang et al., 2017) adjusted weights to ensure positive correlation are used, resulting in activation patterns that may include both positive and negative components. In a previous publication, both cases are considered (Han et al., 2025).

In this study, we proposed a self-supervised learning method. The method presented here is specific to sign statistics, but we note that the method could also be extended to |α|. In that scenario, the top 10% of voxels with the largest |α| values could be assumed to be activated, yielding two decision boundaries in the positive and negative regions. Similar shuffling along these boundaries could then be performed.

### 5.8 Neurological implications

Our findings suggest several neuroscience-relevant implications. First, the activations identified using the proposed nonlinear kernel framework showed substantially greater spatial overlap with gray-matter regions than with the linear model. Because BOLD task activation is physiologically expected to arise predominantly from gray-matter tissue, this improved alignment indicates that the method enhances detection of biologically meaningful activation rather than noise-driven fluctuations. Second, the nonlinear kernels, particularly the mixed hyperbolic tangent kernel, appear better able to model subject-specific variability and nonlinear relationships between neural responses and task conditions. This may be especially important in populations where functional organization is altered or less stereotyped across individuals. Finally, when examining canonical task-related regions of interest, the nonlinear model yielded activation patterns that were more localized to expected functional networks than the linear approach. Together, these results suggest that the proposed method may provide a more neurobiologically faithful representation of task-evoked functional activation and improve sensitivity to subtle but meaningful neural responses.

## 6 Conclusion

In this study, we extend the nonlinear KCCA method from previous work with several key contributions. First, we propose a self-supervised learning method that extracts kernel mappings purely by maximizing shuffling robustness. This method is more efficient, as it does not rely on additional datasets such as resting-state fMRI data, and it provides an alternative way to evaluate performance in the absence of ground truth. Second, we introduce a new back-reconstruction method, which is faster and more accurate than the previous version. In addition, the new method is more robust, with fewer subjects performing below the baseline. Third, we further investigate the kernel selection problem. By evaluating accuracy in fMRI activation detection, augmentation robustness, and eigendecomposition, we show that bounded kernels combining both linear and nonlinear terms outperform other kernels. Empirically, this improvement is attributed to the high noise self-correlation present in traditional kernel methods.

## Figures and Tables

**Appendix Fig. A1.**
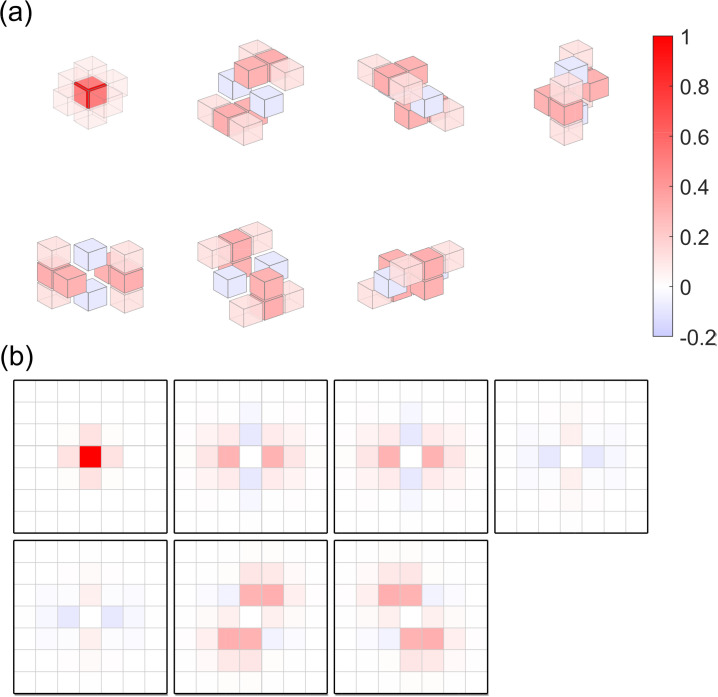
(a) Seven 7×7×7 steerable filters are used for 3D fMRI data with grid size 2 mm. The equal weight sum is equal to a single Gaussian smoothing filter with FWHM = 4 mm. (b) The same as (a) but projected on the x-axis.

**Appendix Fig. A2.**
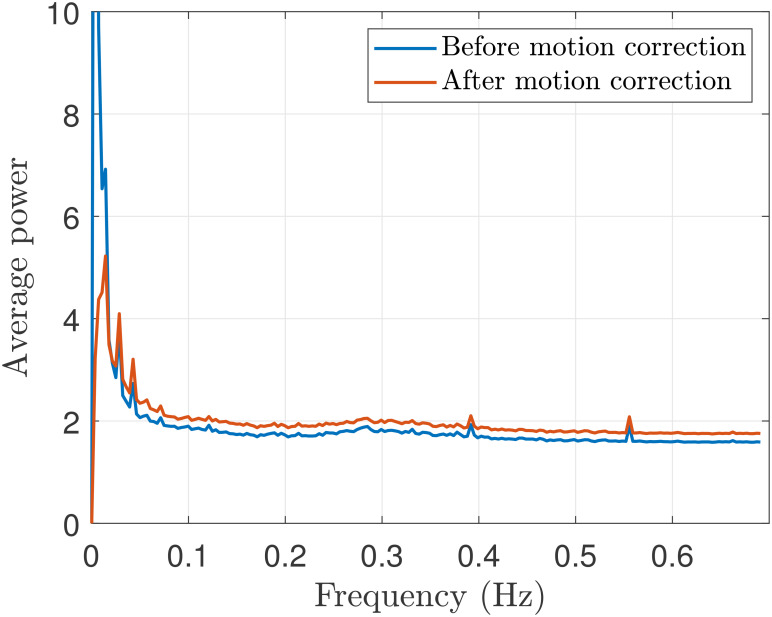
Average power spectral density versus frequency before and after motion correction. Results are averaged across 87 subjects from the HCP dataset.

## Data Availability

The HCP data used in this study are publicly available and can be downloaded through the Human Connectome Project repository. Data access can be requested at https://www.humanconnectome.org/study/hcp-young-adult/data-releases. All the codes used in this study will be publicly available after acceptance at https://github.com/CCLRCBH-BIC.

## Appendix A

### A.1. Steerable filters

Suppose $F_{\text{orig}}$ is the Gaussian filter with FWHM =4 mm used in GLM, and $G_{\text{iso}}$ is defined as a Gaussian function with FWHM equal to half of $F_{\text{orig}}$ (FWHM =2 mm). Let $G_{i}$ (i=1,…,6 ) denote

$$\begin{array}{cc} \begin{array}{c} G_{i}\left(\text{x}\right)=\left(1-G_{\text{iso}}\left(\text{x}\right)\right)\;\left[\left({\hat{\text{n}}}_{i}^{T}\hat{\text{x}}\right)-\frac{1}{6}\right],\\ {\hat{\text{n}}}_{1}=\left(\begin{array}{c} a\\ 0\\ b \end{array}\right),{\hat{\text{n}}}_{2}=\left(\begin{array}{c} -a\\ 0\\ b \end{array}\right),{\hat{\text{n}}}_{3}=\left(\begin{array}{c} b\\ a\\ 0 \end{array}\right),\\ {\hat{\text{n}}}_{4}=\left(\begin{array}{c} a\\ 0\\ b \end{array}\right),{\hat{\text{n}}}_{5}=\left(\begin{array}{c} -a\\ 0\\ b \end{array}\right),{\hat{\text{n}}}_{6}=\left(\begin{array}{c} b\\ a\\ 0 \end{array}\right),\\ a=\frac{2}{\sqrt{10+2\sqrt{5}}},b=\frac{1+\sqrt{5}}{\sqrt{10+2\sqrt{5}}}. \end{array} & \end{array}$$
(A1)

Seven steerable filters, $F_{\text{iso}}$ and $F_{i}$ (i=1,…,6 ), are defined as

$$F_{\text{iso}}\left(\text{x}\right)=G_{\text{iso}}\left(\text{x}\right),F_{i}\left(\text{x}\right)=G_{i}\left(\text{x}\right)F_{\text{orig}}\left(\text{x}\right)\text{ for }i=1,\cdots ,6.$$
(A2)

We can transform those filters to a matrix form A and decompose it to ${\text{A}}^{\left(0,\cdots ,6\right)}$. After expanding the smooth kernel to a 7×7×7 grid, we plotted the result in Appendix Figure A1.

### A.2. Normalization for the kernel method

In Table 3 all the kernels can be generated using the linear or square kernel. We introduce two normalization constants

$$\begin{array}{cc} \begin{array}{cc} N_{\text{Linear}} & =\frac{1}{T^{2}}\sum_{i}\sum_{j}\left|\sum_{p}\left({\tilde{Y}}_{ip}{\tilde{Y}}_{jp}\right)\right|,\\ N_{\text{Square}} & =\frac{1}{T^{2}}\sum_{i}\sum_{j}\left|\sum_{p}{\left({\tilde{Y}}_{ip}-{\tilde{Y}}_{ip}\right)}^{2}\right|. \end{array} & \end{array}$$
(A3)

To simplify notation, we substitute $K_{\text{Y}}$ in the main article by $K_{\text{(Kernel name)}}$ as there is no ambiguity in this context. Together with the normalization, eight kernels are defined as

$$\begin{array}{c} {\left(K_{\text{Linear}}\right)}_{ij}=\frac{1}{N_{\text{Linear}}}\sum_{p}{\tilde{Y}}_{ip}{\tilde{Y}}_{jp},\\ {\left(K_{\text{Parabolic}}\right)}_{ij}={\left(\frac{1}{N_{\text{Linear}}}\sum_{p}{\tilde{Y}}_{ip}{\tilde{Y}}_{jp}+b^{2}\right)}^{2},\\ {\left(K_{\text{Gaussian}}\right)}_{ij}=\text{exp}\left[-\frac{1}{\sigma^{2}}\frac{1}{N_{\text{Square}}}\sum_{p}{\left({\tilde{Y}}_{ip}-{\tilde{Y}}_{jp}\right)}^{2}\right],\\ {\left(K_{\text{Inverse}}\right)}_{ij}=1/\sqrt{\sum_{p}\frac{1}{N_{\text{Square}}}{\left({\tilde{Y}}_{ip}-{\tilde{Y}}_{jp}\right)}^{2}+b^{2}},\\ {\left(K_{\text{Bounded Linear}}\right)}_{ij}=\{\begin{array}{cc} {\left(K_{\text{Linear}}\right)}_{ij} & \text{For}\;{\left(K_{\text{Linear}}\right)}_{ij}<=C,\\ C & \text{For}\;{\left(K_{\text{Linear}}\right)}_{ij}>C. \end{array},\\ {\left(K_{\text{Square}}\right)}_{ij}=\frac{1}{N_{\text{Square}}}\sum_{p}{\left({\tilde{Y}}_{ip}-{\tilde{Y}}_{jp}\right)}^{2},\\ {\left(K_{\text{Tanh}}\right)}_{ij}=\text{tanh}\left(b\sum_{p}\frac{1}{N_{\text{Linear}}}{\tilde{Y}}_{ip}{\tilde{Y}}_{jp}+c\right),\\ {\left(K_{\text{Mixed Tanh}}\right)}_{ij}=\text{tanh}[b_{1}\frac{1}{N_{\text{Linear}}}\sum_{p}{\tilde{Y}}_{ip}{\tilde{Y}}_{jp}+b_{2}\frac{1}{N_{\text{Square}}}\\ \;\;\;\sum_{p}{\left({\tilde{Y}}_{ip}-{\tilde{Y}}_{jp}\right)}^{2}+c]. \end{array}$$
(A4)

### A.3. Proof of Eq. (5)

In Eq. (4), we insert the identity matrix $I=\tilde{Y}{\tilde{Y}}^{T}{\left(\tilde{Y}{\tilde{Y}}^{T}\right)}^{†}$ on the right.

$$\begin{array}{cc} \begin{array}{cc} \text{Y}\alpha -\left(\tilde{\text{Y}}{\tilde{\text{Y}}}^{T}{\left(\tilde{\text{Y}}{\tilde{\text{Y}}}^{T}\right)}^{†}\right)K_{\text{Y}}{\text{v}}_{\text{Y}} & =0\\ \text{Y}\left[\alpha -\text{A}{\tilde{\text{Y}}}^{T}{\left(\tilde{\text{Y}}{\tilde{\text{Y}}}^{T}\right)}^{†}K_{\text{Y}}{\text{v}}_{\text{Y}}\right] & =0. \end{array} & \end{array}$$
(A5)

Let the bracket be equal to 0, then we have

$$\alpha =\text{A}{\tilde{\text{Y}}}^{T}{\left(\tilde{Y}{\tilde{Y}}^{T}\right)}^{†}K_{\text{Y}}{\text{v}}_{\text{Y}}.$$
(A6)

One practical difficulty is that the eigenvector ${\text{v}}_{\text{Y}}$ contains the uncertainty for the sign. Note that the ${\text{X}}_{\text{eff}}$ is represented by the kernel space term $K_{\text{X}}{\text{v}}_{\text{X}}$, then in the kernel space the relationship between $K_{\text{X}}{\text{v}}_{\text{X}}$ and $K_{Y}{\text{v}}_{\text{Y}}$ is maximized. To make the sign consistent, we introduce $s=\text{sign}\left[\text{corr}\left({\text{X}}_{\text{eff}},K_{\text{Y}}{\text{v}}_{\text{Y}}\right)\right]$. Finally,

$$\alpha =s\text{A}{\tilde{\text{Y}}}^{T}{\left(\tilde{\text{Y}}{\tilde{\text{Y}}}^{T}\right)}^{†}K_{\text{Y}}{\text{v}}_{\text{Y}}.$$
(A7)

As the maximum rank for Y is T, removing Y outside the bracket introduces other constraints for the solution. From the relationship between the unknown and the number of equations, the original Eq. (4) only has T constraints. By removing Y we increase the constraints to Q.

### A.4. Proof of Eq. (6)

Starting from Eq. (4), both sides are represented by a vector with length t. For any t we have

$${\text{Y}}_{t}\alpha -{\left(K_{\text{Y}}{\text{v}}_{\text{Y}}\right)}_{t}=0.$$
(A8)

Taken the derivative for $Y_{t}$ on both sides yields

$$\alpha =\frac{\partial {\left(K_{\text{Y}}{\text{v}}_{\text{Y}}\right)}_{t}}{\partial {\text{Y}}_{t}}.$$
(A9)

Practically evaluating this equation has two difficulties. α should satisfy this equation for all times t, and ${\text{v}}_{\text{Y}}$ is the eigenvector corresponding to the largest eigenvalues and it is not differentiable. We take an approximation for this problem by using summation from different times and treat ${\text{v}}_{\text{Y}}$ as a constant during differentiation. Similar to the analyses shown in Section 7.2, we add the sign to remove the uncertainty so that

$$\alpha =s\sum_{t_{1}t_{2}}\frac{\partial {\left(K_{\text{Y}}\right)}_{t_{1}t_{2}}}{\partial {\text{Y}}_{t_{1}}}{\left({\text{v}}_{\text{Y}}\right)}_{t_{2}}.$$
(A10)

From the relationship between the unknown and the number of equations, the original Eq. (4) only has T constraints. By taking derivative, we increased the number of constraints to Q×T . Note that Eq. (A9) is defined for each time t. Finally we reduce the constraints to Q by taking an average of all time points.

### A.5. Proof of equivalence for the linear kernel

For BC 1, we insert $K_{\text{Y}}=\tilde{\text{Y}}{\tilde{\text{Y}}}^{T}$ so that

$$\alpha =s\text{A}{\tilde{\text{Y}}}^{T}{\left(\tilde{\text{Y}}{\tilde{\text{Y}}}^{T}\right)}^{†}\left(\tilde{\text{Y}}{\tilde{\text{Y}}}^{T}\right){\text{v}}_{\text{Y}}=s\text{A}{\text{A}}^{T}{\text{Y}}^{T}{\text{v}}_{\text{Y}}.$$
(A11)

For BC 2, we use the equation from Han et al. (2025) and obtain

$$\alpha =\text{A}\tilde{\alpha },\;\text{and}\;{\tilde{\alpha }}_{p}=\sum_{ij}\frac{\partial {\left(K_{\text{Y}}\right)}_{ij}}{\partial {\tilde{Y}}_{ip}}{\left({\text{v}}_{\text{Y}}\right)}_{j}.$$
(A12)

Then,

$$\begin{array}{cc} \begin{array}{cc} {\tilde{\alpha }}_{p} & =\sum_{ij}\frac{\partial {\left(K_{\text{Y}}\right)}_{ij}}{\partial {\tilde{Y}}_{ip}}{\left({\text{v}}_{\text{Y}}\right)}_{j}\\ & =\frac{1}{N_{\text{linear}}}\sum_{ij}\frac{\partial \sum_{x}{\tilde{Y}}_{ix}{\tilde{Y}}_{jx}}{\partial {\tilde{Y}}_{ip}}{\left({\text{v}}_{\text{Y}}\right)}_{j}\\ & =\frac{1}{N_{\text{linear}}}\sum_{ij}\left[{\tilde{Y}}_{jp}{\left({\text{v}}_{\text{Y}}\right)}_{j}+\delta_{ij}{\tilde{Y}}_{jp}{\left({\text{v}}_{\text{Y}}\right)}_{j}\right]\\ & =\frac{T+1}{N_{\text{linear}}}{\tilde{\text{Y}}}_{p}^{T}\text{v}. \end{array} & \end{array}$$
(A13)

Then, for BC 2 we obtain

$$\alpha =\frac{T+1}{N_{\text{linear}}}\text{A}{\tilde{\text{Y}}}^{T}{\text{v}}_{\text{Y}}=\frac{T+1}{N_{\text{linear}}}\text{A}{\text{A}}^{T}{\text{Y}}^{T}{\text{v}}_{\text{Y}}.$$
(A14)

### A.6. Power analysis before and after motion correction

Six motion parameters from the HCP dataset are denoted as ${\text{z}}_{\text{motion}}\in {ℜ}^{T\times 6}$ (Van Essen et al., 2012). A linear regression model is applied to remove motion-related components:

$${\text{Y}}_{\text{correction}}=\text{Y}-{\text{z}}_{\text{motion}}\text{e},$$
(A15)

where $\text{e}={\left({\text{z}}_{\text{motion}}\right)}^{†}\text{Y}\in {ℜ}^{6\times Q}$ .

Power spectral analysis based on the discrete Fourier transform is then performed on both Y and ${\text{Y}}_{\text{correction}}$ for comparison. Prior to the analysis, each voxel time series is normalized to zero mean and unit variance in the time domain. The resulting Fourier power spectra are shown in Appendix Figure A2, averaged across all 87 subjects in the HCP dataset.

The results show that motion correction reduces power density in the low-frequency range, while increasing power at higher frequencies (>0.05 Hz). This suggests that including motion regressors may introduce additional high-frequency artifacts in the HCP dataset.
